# Natural Variation of *NAR5* Determines Nitrogenase Activity and the Yield in Soybean

**DOI:** 10.1002/advs.202521100

**Published:** 2026-04-14

**Authors:** Chao Ma, Hanyu Zhao, Haojie Feng, Xulun Dong, Lin Chen, Mingliang Yang, Runyi Chen, Chengjun Lei, Chunyan Liu, Qingshan Chen, Dawei Xin, Jinhui Wang

**Affiliations:** ^1^ National Key Laboratory of Smart Farm Technology and System College of Agriculture Northeast Agricultural University Harbin P. R. China; ^2^ Key Laboratory of Soybean Biology in Chinese Ministry of Education College of Agriculture Northeast Agricultural University Harbin P. R. China; ^3^ Suihua Branch of Heilongjiang Academy of Agricultural Sciences Suihua P. R. China

**Keywords:** *Glycine max*, *NAR5*, natural variation, nitrogenase activity

## Abstract

Symbiotic nitrogen fixation (SNF) serves as a vital process through which legumes acquire atmospheric nitrogen, directly influencing plant growth, yield, and soil fertility. Nitrogenase activity represents a key determinant of SNF efficiency, yet only a limited number of genes regulating this process have been identified in soybean nodules. In this study, a genome‐wide association analysis uncovered a major quantitative trait locus (QTL) on chromosome 5, named *NAR5* (*Nitrogenase Activity Related Gene 5*), which governs variation in nitrogenase activity among natural soybean populations under controlled greenhouse conditions. *NAR5* encodes a subtilisin‐like serine protease. Functional characterization demonstrated that *NAR5* overexpression downregulates the transcription of senescence‐associated genes in nodules, thereby sustaining nitrogenase function. Moreover, plants overexpressing *NAR5* exhibited enhanced field performance, with increased yield and improved adaptation to low‐nitrogen conditions. Population analysis revealed that *NAR5* is subject to selection pressure during domestication, and that the elite haplotype *NAR5* ^HapI‐1^ linked to superior nitrogenase activity and greater seed weight has been preferentially incorporated into modern breeding germplasm. In summary, these findings identify *NAR5* as a candidate genetic regulator of SNF efficiency in soybean, providing a promising molecular target for breeding high‐yield, nitrogen‐efficient cultivars.

## Graphical abstract

This study identified *NAR5*, a gene encoding a subtilisin‐like protease, that regulates nitrogenase activity in soybean nodules. Overexpressing *NAR5* delayed nodule senescence, enhancing nitrogenase activity, yield, and low‐nitrogen tolerance. The elite haplotype *NAR5* ^HapI‐1^ linked to superior nitrogenase activity and greater seed weight has been preferentially incorporated into modern breeding germplasm, offering a target for developing high‐yield, nitrogen‐efficient soybean cultivars.

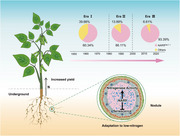

## 1 Introduction

Soybean (*Glycine max* (L.) Merr.) is one of the world's most important oilseed crops and a major source of plant‐derived protein, providing nearly one‐quarter of the global supply of high‐quality vegetable protein [1, 2]. Much like other leguminous species, soybean has the remarkable ability to fix atmospheric nitrogen (N_2_) through the formation of root nodules in association with symbiotic rhizobia, a process known as symbiotic nitrogen fixation (SNF). Because crop productivity is heavily dependent on nutrient availability, nitrogen deficiency is often a major factor limiting yield potential. In soybean, SNF contributes approximately 60%–70% of the total nitrogen required for growth and development, underscoring the pivotal role of the soybean–rhizobium symbiosis in sustaining yield and enhancing soil fertility [3, 4]. Over the course of roughly 5000 years of domestication from its wild progenitor (*Glycine soja* Sieb. & Zucc.), many favorable allelic variants that influence SNF efficiency have been consciously or inadvertently selected to improve adaptability to nutrient‐poor soils as well as to optimize yield and quality. Nevertheless, despite advances in genomics and breeding, relatively few superior alleles directly associated with nitrogenase activity in soybean have been identified and characterized to date.

The initiation of symbiotic interaction between legumes and rhizobia begins with precise molecular recognition between the plant host and the bacterial partner, ultimately leading to the activation of bacterial nitrogenase enzymes, which mark the onset of mature nitrogen‐fixing symbiosis [5, 6]. Within symbiosomes, rhizobia differentiate into nitrogen‐fixing bacteroids, which convert atmospheric nitrogen into ammonia and release it into the plant cytoplasm [7]. Leghemoglobins play a crucial role in maintaining optimal oxygen concentrations in nodules, creating a microaerobic environment that supports nitrogenase gene expression and activity [8, 9, 10]. As nodules age, degradation of leghemoglobin heme groups into biliverdin and bilirubin leads to a visible color change from red to green, a physiological marker correlated with the decline in nitrogenase activity and the onset of nodule senescence [11, 12, 13]. Ammonia transport, assimilation, and subsequent synthesis of organic nitrogen compounds are fundamental processes governing root nodule function [5]. Efficient SNF also relies on the coordinated function of various nitrate and ammonium or sucrose transporters. Mutations in transporter genes such as *LjAMT1;1*, *LjNPF8.6*, and *MtSWEET11* have been shown to reduce nitrogenase activity, highlighting their importance in nitrogen acquisition and metabolism [14, 15, 16]. In addition, metal ions act as essential cofactors for numerous metalloenzymes that participate in SNF [17, 18]. Metal ion transporters and their transcriptional regulators modulate the biosynthesis and stability of iron‐containing hemoglobins, (Fe–Mo)‐ and (Fe–S)‐nitrogenases, and (Cu–Fe)‐cytochromes in nodules, thereby ensuring sustained nitrogenase functionality [19, 20, 21]. Environmental conditions also strongly affect nitrogenase activity. Variations in soil pH, temperature, water availability, and nitrogen content can markedly influence the efficiency of biological nitrogen fixation [22, 23, 24, 25, 26]. Moreover, the presence of soil pollutants or imbalances in mineral nutrients may disrupt the delicate symbiotic equilibrium between legumes and rhizobia, further diminishing nitrogenase performance [27, 28, 29, 30]. Collectively, these multifaceted biological and environmental interactions illustrate the inherent complexity of SNF and underscore the challenges of identifying molecular targets or agronomic strategies capable of optimizing nitrogenase efficiency in leguminous crops.

Subtilases (SBTs) are a family of serine proteases that are widely distributed across eukaryotes [31]. Although plant SBTs share structural similarities with their bacterial and mammalian counterparts, evolutionary divergence has endowed them with distinct and specialized functions in plants [31, 32, 33]. These enzymes are now recognized as key regulators involved in diverse developmental and physiological processes [34]. However, the extensive expansion and functional diversification of the SBT gene family have made it difficult to precisely define their individual biological roles and substrate specificities [35]. Members of the SBT family participate in both broad protein degradation and highly specific processing proteases events, thereby regulating diverse developmental and physiological processes, including embryogenesis, seed maturation and germination, aging, and responses to environmental stimuli [31, 36]. Increasing evidence indicates that certain SBTs exhibit nodule‐specific or symbiosis‐related expression patterns, suggesting potential roles in SNF regulation [37, 38, 39]. Nevertheless, the molecular mechanisms by which SBTs contribute to symbiosis remain poorly defined, especially in soybean, where such studies are still limited. Elucidating the molecular functions of SBTs in soybean is therefore critical for advancing the current understanding of SNF and breeding cultivars with enhanced nitrogen‐fixing capabilities.

Northeast China is the largest main production area of soybeans in China, accounting for nearly half of the total national output (http://www.moa.gov.cn/). To investigate the genetic determinants underlying SNF efficiency, we analyzed 309 soybean germplasm accessions originating mainly from Heilongjiang, Jilin, Liaoning, and Inner Mongolia. Through integrated QTL mapping of recombinant inbred lines (RILs) and genome‐wide association study (GWAS) approaches, we identified *NAR5*, a subtilisin‐like protease gene, as a major determinant of nitrogenase activity. Natural polymorphisms within its promoter region were found to modulate gene expression and nodule senescence timing. Overexpression of *NAR5* significantly increased nitrogenase activity and enhanced grain yield, particularly under reduced nitrogen fertilization. Collectively, our results illuminate the genetic foundation of efficient SNF and nitrogen utilization in soybean and introduce novel allelic variants that can be leveraged to develop high‐yielding, nitrogen‐efficient cultivars.

## 2 Results

### 2.1 Identification of Nitrogenase Activity Loci in Soybean

**FIGURE 1:**
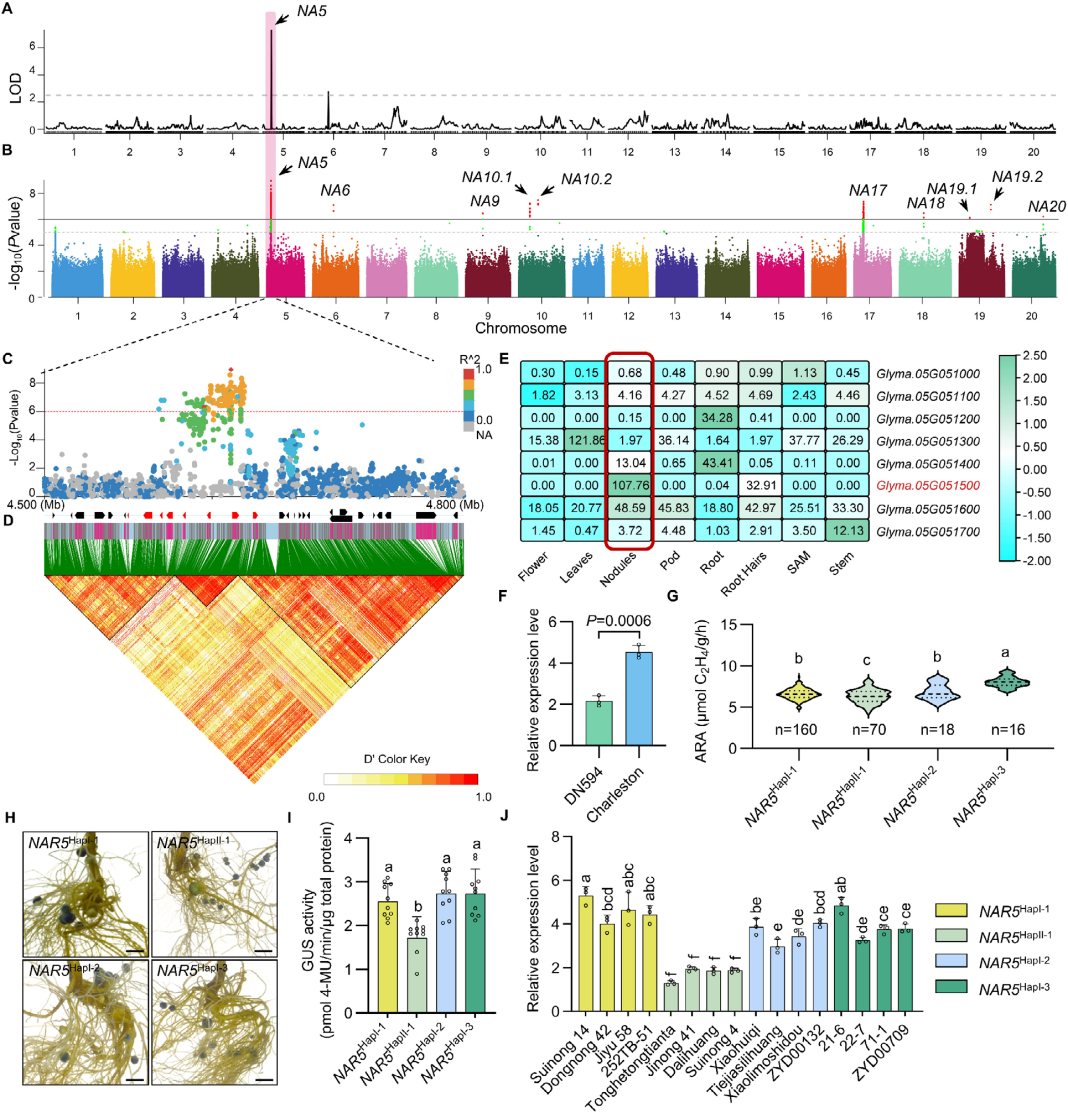
Nitrogenase activity QTL mapping and GWAS, and haplotype analysis of *NAR5*. (A) Whole‐chromosome scan for nitrogenase activity‐related QTLs in root nodules from a soybean recombinant inbred line (RIL, *n* = 147) population from Dongnong594 × Charleston, with a LOD threshold of 2.5. (B) GWAS Manhattan plot for a nitrogenase activity‐related GWAS in root nodules from 309 soybean accessions. (C) Genome‐wide Manhattan plot corresponding to the 4.500–4.800 Mb region of chromosome 5. The threshold signal level is marked with a dashed line. (D) Linkage disequilibrium (LD) plot highlighting SNPs in the 4.500–4.800 Mb association block. Black and red arrows respectively denote genes and the candidate gene. The gradient from white to red is proportional to the degree of LD, as determined using pairwise r^2^ values and local LD boundaries defined by the *r* ^2^ > 0.2. (E) Candidate gene expression heatmap. Candidate gene expression levels (transcripts per million kilobases, TPM) in the identified locus are represented with a color gradient from blue to green using TPM data from ePlant (https://bar.utoronto.ca/eplant_soybean/). (F) Relative *NAR5* expression in DN594 and Charleston nodules. Data are means ± SD (*n* = 3 biological replicates). Data were analyzed using Student's *t*‐tests. (G) Haplotype analysis of *NAR5* ^HapI‐1^ (*n* = 160 accessions), *NAR5* ^HapII‐1^ (*n* = 70 accessions), *NAR5* ^HapI‐2^ (*n* = 18 accessions), and *NAR5* ^HapI‐3^ (*n* = 16 accessions). Data were analyzed using one‐way ANOVAs and Tukey's multiple‐comparison test (*p* < 0.05), and different lowercase letters indicate significant differences among groups. (H) Promoter activity analysis for each haplotype (*NAR5* ^HapI‐1^, *NAR5* ^HapII‐1^, *NAR5* ^HapI‐2^, and *NAR5* ^HapI‐3^) based on evaluation of a 3‐kb sequence upstream of the translation initiation site fused to the β‐glucuronidase (*GUS*) reporter gene in soybean hairy roots. Scale bar: 5 mm. (I) Analysis of GUS enzyme activity levels in (H). Data are means ± SD (*n* = 10 biological replicates) and were compared using one‐way ANOVAs with Tukey's multiple‐comparison test (*p* < 0.05), different lowercase letters indicate significant differences among groups. (J) Relative *NAR5* expression levels in root nodules from randomly selected soybean accessions harboring different haplotypes. Data are means ± SD (*n* = 3 biological replicates) and were compared using one‐way ANOVAs with Tukey's multiple‐comparison test (*p* < 0.05), different lowercase letters indicate significant differences among groups.

To uncover the genetic loci influencing nitrogenase activity, we assessed a RIL population (*n* = 147) derived from a cross between Dongnong594 and Charleston, two parental lines displaying pronounced differences in nitrogenase activity (Figure S1). In parallel, we performed a GWAS using 309 soybean accessions (123 cultivars, 90 landraces, and 96 wild types; Figure S2A−D) from Northeast China (Heilongjiang, Jilin, Liaoning, and Inner Mongolia), employing 6 987 829 SNP markers with a minor allele frequency >5%. This combined strategy enabled the identification of genetic regions associated with nitrogenase activity variation in soybean. QTL analysis of the RIL population identified two loci linked to nitrogenase activity, while the GWAS, using a mixed linear model (MLM), detected ten significant loci across the genome (Figure 1A,B, Figure S2E, and Tables S1,S2). These loci were designated Nitrogenase Activity 5 through 20 (*NA5*–*NA20*).

To reduce the likelihood of false positives inherent to single‐model analyses, we further employed GLM and FarmCPU models, which consistently confirmed the *NA5* and *NA17.2* loci identified by the MLM (Figure 1B, Figure S3A−C). Both QTL and GWAS analyses converged on a 101.1‐kb candidate interval on chromosome 5 corresponding to the *NA5* locus (Figure 1C,D, Figure S3D). Within this genomic region, eight protein‐coding genes were annotated in the Wm82 reference genome, among which *Glyma.05G051500* exhibited notably higher expression in root nodules, which are the organ most closely linked to nitrogenase activity (Figure 1E). Moreover, expression of *Glyma.05G051500* significantly differed between the nodules of Dongnong 594 and Charleston (Figure 1E). Based on these findings, we designated *Glyma.05G051500* as *NAR5* (*Nitrogenase Activity Related Gene 5*) for further functional analysis.

*NAR5* encodes a subtilase‐family protein, and phylogenetic reconstruction coupled with sequence alignment revealed that its closest homologs (sharing >60% amino acid identity) are predominantly conserved among leguminous species capable of symbiotic nitrogen fixation (SNF) (Figures S4 and S5), consistent with a role for *NAR5* in symbiotic processes. Haplotype and linkage disequilibrium analyses of the 3‐kb upstream region and coding sequence identified 17 SNPs in the promoter and 6 SNPs within exons that lead to amino acid substitutions (Figure S6A–C). Notably, three polymorphisms located at −1469, −271, and −237 bp relative to the translation start site altered predicted cis‐acting regulatory elements within the promoter (Figure S6D). These SNPs displayed significant associations with nitrogenase activity (Figure S6B). The gene region exhibited two major linkage blocks, with the second—spanning the proximal promoter and coding sequence—containing SNPs exceeding the genome‐wide significance threshold (*P* = 10^6^; Figure S6B). Based on these high‐impact variants and linkage structures, four primary haplotypes were defined: *NAR5* ^HapI‐1,2,3^ and *NAR5* ^HapII‐1^, grouped into two haplotype classes. Comparative analysis across the 309 resequenced accessions demonstrated that accessions carrying *NAR5* ^HapI^ exhibited substantially greater nitrogenase activity than those with *NAR5* ^HapII^ (Figure 1F). These results establish *NAR5* as a pivotal gene modulating nitrogenase activity and symbiotic efficiency in soybean.

### 2.2 Natural Variation in the Promoter of *NAR5* Primarily Influences Nitrogenase Activity

To explore how sequence polymorphisms in *NAR5* influence nitrogenase activity, we first analyzed nucleotide variations across its coding region. Although several SNPs produced amino acid substitutions (K275E, K424R, E429R, K430R, N544D, and A546D), none occurred within the conserved catalytic residues of the subtilase domain (Figure S7A). Structural modeling using AlphaFold3 predicted that these substitutions did not disrupt either the local secondary structure or the overall tertiary conformation surrounding the altered residues (Figures S7B,C). Structural superimposition of the *NAR5* ^HapI^ and *NAR5* ^HapII^ proteins likewise revealed no notable differences in folding, except for minor variations in the signal peptide region (Figure S7D). Because signal peptides govern protein localization, we examined subcellular trafficking patterns for both haplotypes and found that each localized exclusively to the endoplasmic reticulum (Figure S7E).

To directly assess whether coding‐sequence variation in *NAR5* affects nitrogenase activity, we generated transgenic DN50 hairy roots expressing *NAR5^DN50^_pro_: GFP*, *NAR5^DN50^_pro_:NAR5^HapI^‐GFP* or *NAR5^DN50^_pro_:NAR5^HapII^‐GFP* constructs (Figure S7F−H). Nitrogenase activity, assessed 28 days post‐inoculation, was significantly enhanced in nodules overexpressing *NAR5* compared with the control (Figure S7I). However, no significant difference was observed between *NAR5* ^HapI^ and *NAR5* ^HapII^ overexpressing roots (Figure S7I). These findings collectively indicate that natural SNP variation within the *NAR5* coding region does not substantially alter its functional effect on nitrogenase activity.

We next examined whether promoter polymorphisms account for variation in *NAR5* expression and nitrogenase activity among soybean accessions. Transgenic hairy roots harboring β‐glucuronidase (*GUS*) under the control of promoters from four distinct *NAR5* haplotypes revealed markedly stronger promoter activity in roots carrying *NAR5* ^HapIs^ constructs than in those containing *NAR5* ^HapII^ (Figure 1H,I). These results were corroborated by luciferase reporter assays in tobacco leaves, which similarly showed higher activity for *NAR5* ^HapI‐1, 2, and 3^ (Figure S8A,B). In addition, qRT‐PCR quantification of four randomly selected soybean accessions from each haplotype confirmed significantly lower *NAR5* transcript abundance in *NAR5* ^HapII‐1^ accessions compared with *NAR5* ^HapI^ accessions, consistent with differences in nitrogenase activity among the same accessions (Figure 6I, Figure S8C). Across all samples, *NAR5* expression levels were positively correlated with nitrogenase activity (*R* = 0.68, *p* = 0.0036; Figure S8D). Collectively, these data demonstrate that natural variation in the *NAR5* promoter, rather than in its coding sequence, is the principal determinant of nitrogenase activity differences among soybean lines.

### 2.3 *NAR5* Haplotype Variation Affects Nodule Nitrogenase Activity and Senescence in Infected Cells

**FIGURE 2:**
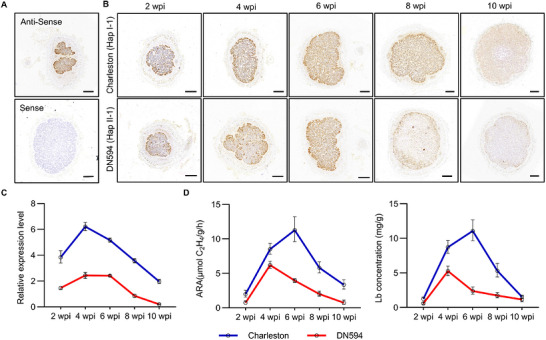
*NAR5* expression patterns in the root nodules of the Charleston and DN594 soybean cultivars. (A) *NAR5* anti‐sense and sense probe in situ hybridization assay in Charleston root nodules at 4 weeks post‐inoculation (4 wpi). (B) *NAR5* spatial expression analysis in Charleston (*NAR5* ^HapI‐1^) and DN594 (*NAR5* ^HapII‐1^) nodules at 2, 4, 6, 8, and 10 wpi. Scale bars: 500 µm in (A) and (B). Representative results are shown from at least 5 independent embedded samples. (C–E) Root nodule *NAR5* expression and related physiological traits in Charleston and DN594. Relative *NAR5* expression (C), nitrogenase activity (D), and leghemoglobin content (E) were assessed at 2, 4, 6, 8, and 10 wpi. Blue and Red lines respectively, correspond to cultivars Charleston and DN594. Data are means ± SD (*n* = 3 biological replicates).

Previous results indicated that *NAR5* is predominantly expressed in soybean nodules (Figure 1D). To better resolve its spatial expression pattern, we analyzed stereo‐seq and snRNA‐seq datasets from the SoyOmics database (https://ngdc.cncb.ac.cn/soyomics/index) [40], which revealed strong *NAR5* expression specifically within infected nodule cells (Figure S9A,B). To further examine how promoter SNPs affect expression dynamics and nitrogenase activity, we performed RNA in situ hybridization in nodules of Charleston (*NAR5* ^HapI‐1^) and DN594 (*NAR5* ^HapII‐1^) plants at different developmental stages. The results were consistent with omics data, showing high *NAR5* expression in infected cells that declined markedly during nodule senescence, aligning with previous transcriptomic reports (Figure 2A,B). Because nodule aging begins centrally, the loss of *NAR5* signal in the nodule core provides additional evidence linking its expression to senescence progression. Notably, DN594 (*NAR5* ^HapII‐1^) exhibited premature senescence relative to Charleston, which likely accounts for its reduced nitrogenase activity (B).

We next quantified temporal changes in *NAR5* expression and nitrogenase activity between these two varieties from 2 to 10 weeks post‐inoculation (wpi). Across all time points, Charleston maintained higher *NAR5* transcript levels and nitrogenase activity than DN594 (Figure 2C). After 4 wpi, nitrogenase activity began to decline more rapidly in DN594, coinciding with a sharper reduction in *NAR5* expression (Figure 2C,D). Because efficient nitrogenase function requires sufficient leghemoglobin to sustain microaerobic conditions, we also measured leghemoglobin content. In agreement with the molecular data, DN594 accumulated significantly less leghemoglobin than Charleston, particularly after 4 wpi (Figure 2E).

These findings collectively establish that *NAR5* expression within infected nodule cells is closely linked to nitrogenase activity performance. The *NAR5* ^HapII^ allele, as exemplified by DN594, is associated with premature decline in *NAR5* transcription, reduced leghemoglobin accumulation, and early onset of nodule senescence, explaining its diminished nitrogenase activity.

### 2.4 *NAR5* Positively Regulates Nitrogenase Activity in Soybean Nodules

**FIGURE 3:**
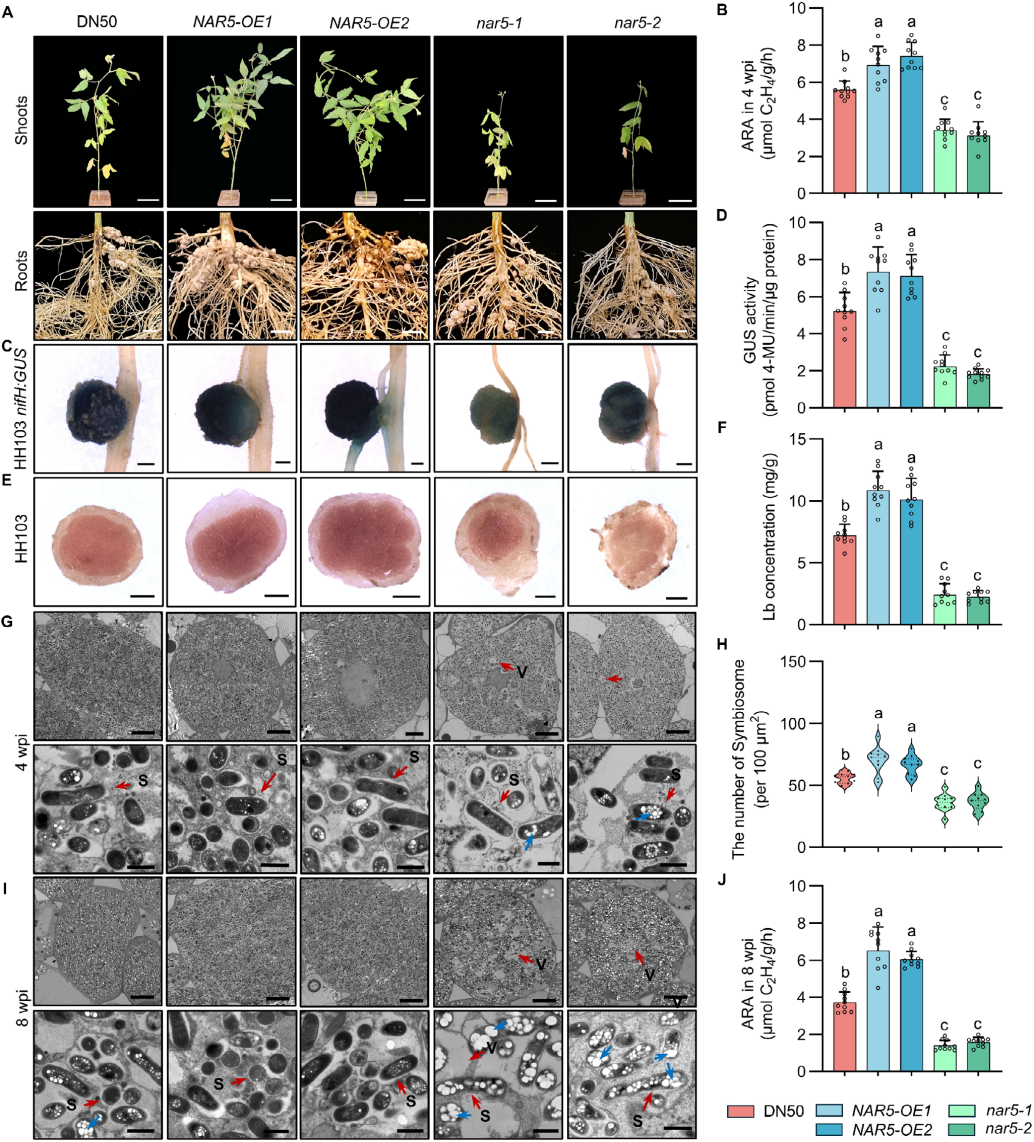
*NAR5* functions as a positive regulator of nitrogenase activity in soybean root nodules. (A) Shoot and root phenotypes for wild‐type (DN50), *NAR5*‐overexpressing (*NAR5‐OE1* and *NAR5‐OE2*), and *nar5* mutant plants (*nar5‐1* and *nar5‐2*) at 4 weeks post‐inoculation (wpi) with *S. fredii* HH103. Shoots: Scale bars = 7.5 cm; Roots: Scale bars = 5 mm. (B) Analysis of the nitrogenase activity in (A). Data are means ± SD (*n* = 10 biological replicates) and were compared using one‐way ANOVAs with Tukey's multiple‐comparison test (*p* < 0.05), different lowercase letters indicate significant differences among groups. (C) Images of mature root nodules from DN50, *NAR5‐OEs*, and *nar5* mutant plants inoculated with HH103 *nifH:GUS* at 4 wpi. Scale bars = 1 mm. Representative images are shown from at least 5 independent embedded samples. (D) Analysis of GUS enzyme activity levels in (C). Data are means ± SD (*n* = 10 biological replicates) and were compared using one‐way ANOVAs with Tukey's multiple‐comparison test (*p* < 0.05), different lowercase letters indicate significant differences among groups. (E) Cross‐sections of mature nodules from DN50, *NAR5‐OEs*, and *nar5* mutant plants. Scale bars = 1 mm. Representative images are shown from at least 5 independent embedded samples. (F) Leghemoglobin (Lb) content in DN50, *NAR5‐OEs*, and *nar5* mutant plant root nodules at 4 wpi. Data are means ± SD (*n* = 10 biological replicates) and were compared using one‐way ANOVAs with Tukey's multiple‐comparison test (*p* < 0.05), different lowercase letters indicate significant differences among groups. (G) Transmission electron micrograph (TEM) images of root nodules from DN50, *NAR5‐OEs*, and *nar5* mutant plants at 4 wpi. V: Lytic vacuolar compartments; S: Symbiosome. Poly‐β‐hydroxybutyrate (PHB) is marked with blue arrows. Upper panel: Scale bar = 10 µm; Lower panel: Scale bar = 1 µm. (H) Quantification of symbiosomes per 100 µm^2^ in TEM images. Data are means ± SD (*n* = 10 biological replicates) and were compared using one‐way ANOVAs with Tukey's multiple‐comparison test (*p* < 0.05), different lowercase letters indicate significant differences among groups. (I) TEM images of nodules from DN50, *NAR5‐OEs*, and *nar5* mutant plants at 8 wpi. V: Lytic vacuolar compartments; S: Symbiosome, marked with red arrows. PHB is marked with blue arrows. Upper panel: Scale bar = 10 µm; Lower panel: Scale bar = 1 µm. (J) Analysis of nitrogenase activity in nodules from DN50, *NAR5‐OEs*, and *nar5* mutant plants at 8 wpi. Data are means ± SD (n = 10 biological replicates) and were compared using one‐way ANOVAs with Tukey's multiple‐comparison test (*p* < 0.05), different lowercase letters indicate significant differences among groups.

To directly examine the regulatory role of *NAR5* in nitrogenase activity, we established *NAR5*‐overexpressing lines (*NAR5‐OE1* and *NAR5‐OE2*) on the DN50 background (*NAR5* ^Hap1‐1^ haplotype), and CRISPR/Cas9‐induced knockout mutants (Figure S10A,B). Both overexpression lines displayed a pronounced increase in nitrogenase activity, whereas *nar5* knockout plants exhibited a significant reduction (Figure 3A,B). Consistent results were obtained from stable RNA interference (RNAi) lines, which showed markedly lower nitrogenase activity compared with the wild type (Figure S11A–C). Importantly, introducing *NAR5* driven by its native promoter (from *NAR5^DN50^*) into *nar5* hairy roots fully restored nitrogenase activity (Figure S12A–E), confirming that the observed phenotype results specifically from the loss of NAR5 function.

To visualize nitrogenase activity in situ, we employed a *S. fredii* HH103 strain carrying a *nifH_pro_* reporter construct fused to *GUS*, in which the *nifH* promoter drives expression of a key nitrogenase structural component (nifHDK) [41]. Nodules from *NAR5‐OE1* and *NAR5‐OE2* plants exhibited strong GUS staining, whereas *nar5* nodules displayed faint signals (Figure 3C). Quantitative assays of GUS activity further confirmed the highest enzymatic activity in NAR5‐overexpressing nodules (Figure 3D).

Morphological analyses also revealed clear physiological differences among genotypes. Cross‐sections of nodules from *NAR5‐OE* plants showed a deep red coloration characteristic of high leghemoglobin content, whereas *nar5* nodules appeared light brown, indicative of heme degradation and nodule senescence (Figure 3E). Direct measurements corroborated these observations, with leghemoglobin levels highest in *NAR5‐OE*, intermediate in wild type, and lowest in *nar5* mutants (Figure 3F). Together, these results demonstrate that *NAR5* functions as a positive regulator of nitrogenase activity in soybean nodules, and that enhancing its expression can effectively promote sustained nitrogen fixation and delayed nodule senescence.

### 2.5 *NAR5* Modulates Nitrogenase Activity by Regulating Nodule Senescence

To elucidate how *NAR5* influences nitrogenase activity at the cellular level, we conducted transmission electron microscopy (TEM) analyses of the largest mature nodules located at the base of the primary root in DN50, *NAR5‐OE1*, and *nar5‐1* plants, given the understanding that nodulation proceeds heterogeneously across roots. In comparison with the wild type, *NAR5‐OE1* nodules displayed a greater density of symbiosomes within infected cells, whereas *nar5‐1* plants exhibited a pronounced reduction (Figure 3G,H). Moreover, symbiosomes in *nar5‐1* nodules showed clear structural aberrations, including extensive accumulation of poly‐β‐hydroxybutyrate (PHB) granules, enlarged lytic vacuoles, and disruption of symbiosome membranes (Figure 3G). Similar ultrastructural defects were observed in *NAR5*‐silenced soybean lines (Figure S11D).

Given the concurrent decreases in leghemoglobin content, the PHB accumulation, and the damage to symbiosome membranes, all of which are hallmark features of senescing nodules [4, 13, 42, 43], we hypothesized that *NAR5* deficiency accelerates nodule senescence, thereby diminishing nitrogenase activity, while *NAR5* overexpression confers delayed senescence. To test this hypothesis, nitrogenase activity and leghemoglobin concentrations were quantified in DN50, *NAR5‐OE1*, and *nar5‐1* plants across five developmental stages from 2 to 10 weeks post‐inoculation (wpi). As previously observed for the *NAR5* ^HapI−1^ haplotype, nitrogenase activity in DN50 nodules began to decline after 6 wpi (Figure S13A). In contrast, *NAR5‐OE1* plants maintained elevated nitrogenase activity and postponed senescence, while *nar5‐1* mutants exhibited premature declines in both nitrogenase activity and leghemoglobin content starting at 4 wpi (Figure S13A,B).

By 8 wpi, nodules from *NAR5‐OE1* remained metabolically active and free of visible senescence, unlike the wild‐type and *nar5‐1* nodules, which had senesced (Figure S13A). To further investigate these temporal differences, we examined cellular morphology in mature nodules at 4 and 8 wpi. Toluidine blue‐stained sections of *nar5‐1* nodules at 4 wpi revealed cytoplasmic vesiculation and extensive vacuolation (Figure S14A), which became more severe by 8 wpi and coincided with a clear reduction in bacteroid abundance (Figure S14B). TEM confirmed intensified senescence in *nar5‐1* nodules at 8 wpi, characterized by extensive PHB deposition and degradation of symbiosome membranes (Figure 3I). Conversely, *NAR5‐OE1* nodules exhibited minimal PHB accumulation and maintained intact symbiosomes at both time points, consistent with their sustained nitrogenase activity (Figure 3I,J).

To further substantiate the role of *NAR5* in suppressing nodule senescence, we assessed programmed cell death using TUNEL assays on fresh nodule sections co‐stained with DAPI and fluorescently labeled dUTP. In line with the structural and biochemical observations, *nar5‐1* nodules exhibited strong TUNEL fluorescence by 4 wpi, which intensified by 8 wpi (Figure S14C,D). By contrast, *NAR5‐OE1* nodules showed markedly weaker TUNEL signals than DN50 at 8 wpi (Figure S14D). These data are entirely consistent with the nitrogenase and ultrastructural phenotypes (Figure 3J), demonstrating that *NAR5* sustains nitrogenase activity primarily by delaying nodule senescence in soybean.

### 2.6 Overexpression of *NAR5* is Associated With Altered Expression of Senescence‐Associated Genes in Nodules

**FIGURE 4:**
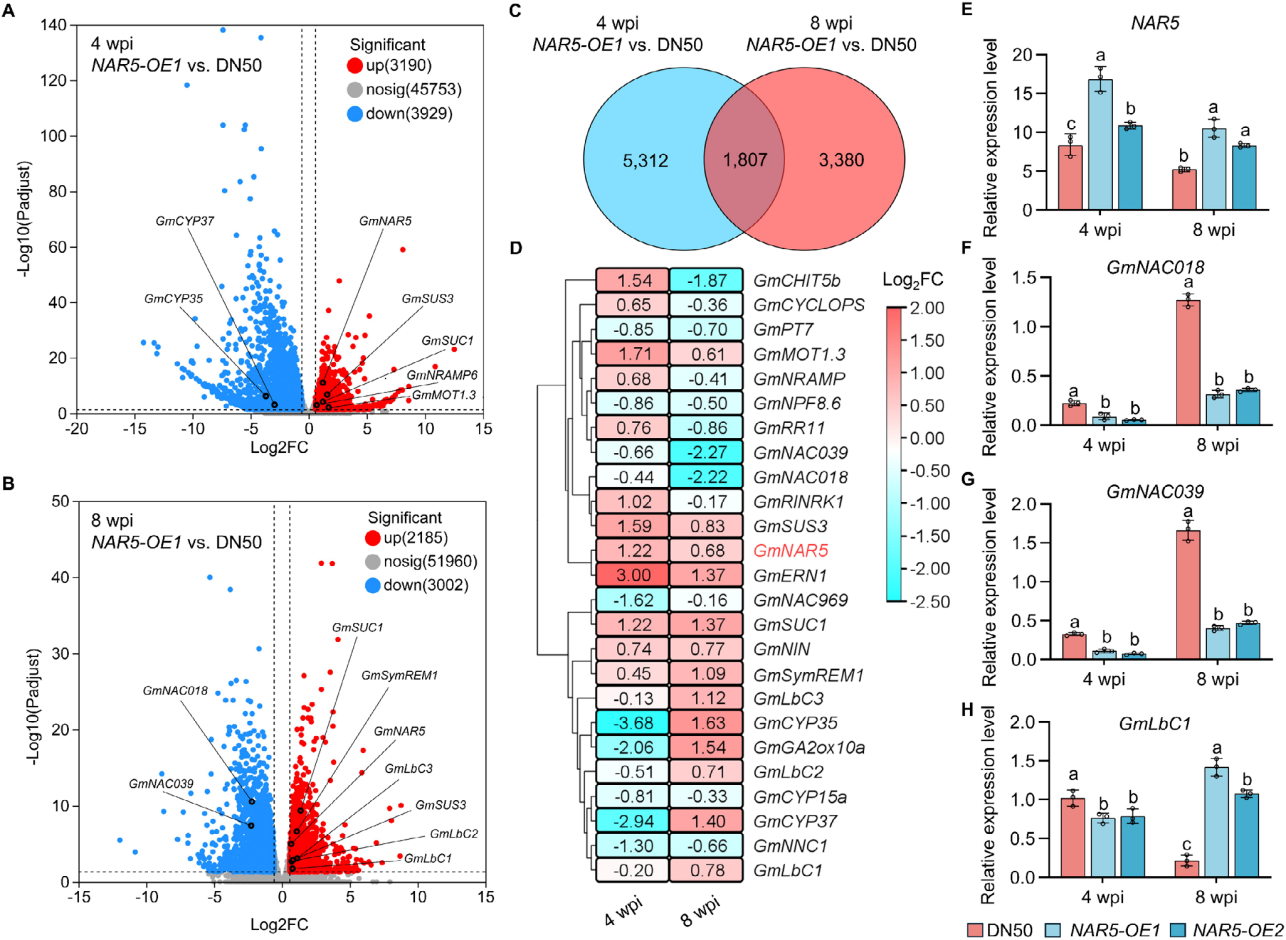
Modulation of host symbiosis and senescence gene expression by *NAR5*. (A, B) Volcano plots showing differentially expressed genes (DEGs) identified in *NAR5‐*overexpressing root nodules (*NAR5‐OE1*) compared to wild‐type DN50 control nodules at 4 weeks post‐inoculation (wpi) (A) and 8 wpi (B). (C) Venn diagram showing overlapping and unique DEGs identified for the indicated pairwise comparisons. (D) Heat maps depicting symbiosis‐associated DEGs identified when comparing *NAR5‐OE1* and DN50 nodules at 4 or 8 wpi. The scale bar corresponds to log_2_(Fold Change) in gene expression. (E–H) Relative *NAR5* (E), *GmNAC018* (F), *GmNAC039* (G), and *GmLbC1* (H) expression in DN50 and *NAR5*‐overexpressing lines (*NAR5‐OE1* and *NAR5‐OE2*) at 4 and 8 wpi. *GmUKN1* was used for normalization. Data are means ± SD (*n* = 3 biological replicates), and were compared using one‐way ANOVAs with Tukey's multiple‐comparison test (*p* < 0.05), different lowercase letters indicate significant differences among groups.

To elucidate the molecular mechanisms by which *NAR5* enhances nitrogenase activity and suppresses senescence, we examined transcriptional changes in response to *NAR5* overexpression. Comparative transcriptomic profiling was performed on nodules from *NAR5‐OE1* and wild‐type DN50 plants at 4 wpi (representing peak nitrogenase activity) and 8 wpi (a stage associated with nodule senescence). At 4 wpi, *NAR5* overexpression resulted in 3190 genes being upregulated and 3929 being downregulated relative to DN50 (Figure 4A, Table S4). At 8 wpi, 2,185 genes were upregulated and 3002 downregulated (Figure 4B, Table S5), with 1807 differentially expressed genes (DEGs) shared between both stages (Figure 4C). GO enrichment analysis indicated that these common DEGs were significantly enriched in biological processes related to responses to chitin, jasmonic acid, and fatty acid metabolism, which are pathways that are closely tied to plant‐microbe interactions and metabolic regulation (Figure S15A). KEGG analysis further revealed notable enrichment in phenylpropanoid biosynthesis, starch and sucrose metabolism, and cysteine and methionine metabolism (Figure S15B). Together, these analyses suggest that *NAR5* overexpression induces extensive transcriptional reprogramming associated with metabolic remodeling, stress response modulation, and immune signaling. Moreover, the DEGs identified in *NAR5‐OE1* nodules were inversely correlated with those observed in senescent versus healthy nodules in previous transcriptomic studies (Pearson's *R* < 0, *p* < 2.2 × 10^−16^; Figure S16A–D) [13]. This correlation is consistent with the observed physiological role of NAR5 in delaying nodule senescence and sustaining nitrogenase activity.

Overexpression of *NAR5* was associated with transcriptomic reprogramming of symbiotic nitrogen fixation and nodule senescence pathways. At 4 wpi, compared to DN50, nodules overexpressing *NAR5* showed significant suppression of *GmNAC039*, *GmNAC018*, *GmCYP35*, and *GmCYP37*, key negative regulators of nitrogenase activity and promoters of nodule senescence [13], correlating with the observed increase in nitrogenase activity (Figure 4D). This repression of *GmNAC039* and *GmNAC018* was maintained at 8 wpi and confirmed by qRT‐PCR (Figure 4D–G). To further investigate the association of *NAR5* on the expression patterns of *GmNAC039* and *GmNAC018* across different developmental stages of soybean nodules, we subsequently quantified the transcript levels of *GmNAC039* and *GmNAC018* in nodules collected at 2, 4, 6, 8, and 10 wpi from DN50, *NAR5*‐overexpressing line (*NAR5‐OE1*) and *nar5* mutant plants (*nar5‐1*). The results revealed that, throughout nodule development, the expression of both *GmNAC039* and *GmNAC018* was significantly higher in the *nar5‐1* mutant compared with DN50, whereas their expression was reduced in the *NAR5‐OE1* (Figure S17A). These findings further support the role of NAR5 in repressing the transcription of *GmNAC039* and *GmNAC018*. Subsequently, to evaluate the spatiotemporal expression patterns of *NAR5*, *GmNAC039*, and *GmNAC018*, we analyzed publicly available stereo‐seq and snRNA‐seq datasets from the SoyOmics database (https://ngdc.cncb.ac.cn/soyomics/index) [40]. The data indicated that *GmNAC039* and *GmNAC018* are predominantly expressed in the outer cortex cells and are barely detectable in the symbiotic zone (Figure S17B,C). This spatial separation suggests that NAR5 might indirectly influence *GmNAC039* and *GmNAC018* expression, though the precise mode of regulation remains unclear. Next, expression analysis of the leghemoglobin gene *GmLbC1* revealed a transient downregulation at 4 wpi in *NAR5*‐overexpressing nodules (Figure 4D). However, this contrasted with elevated leghemoglobin protein levels (Figure 3F), suggesting potential post‐transcriptional compensation that maintains leghemoglobin homeostasis. At 8 wpi, *GmLbC1* expression was significantly upregulated in *NAR5‐OEs* (Figure 4D,H), aligning with both sustained nitrogenase activity and the attenuation of senescence‐related transcriptomic signatures. Taken together, our findings indicate that *NAR5* enhances nitrogenase activity and delays nodule senescence. This effect may involve partly the indirect transcriptional repression of key senescence‐promoting regulators, including *GmNAC039* and *GmNAC018*.

### 2.7 *NAR5^HapI‐1^* Underwent Selection during Soybean Domestication

**FIGURE 5:**
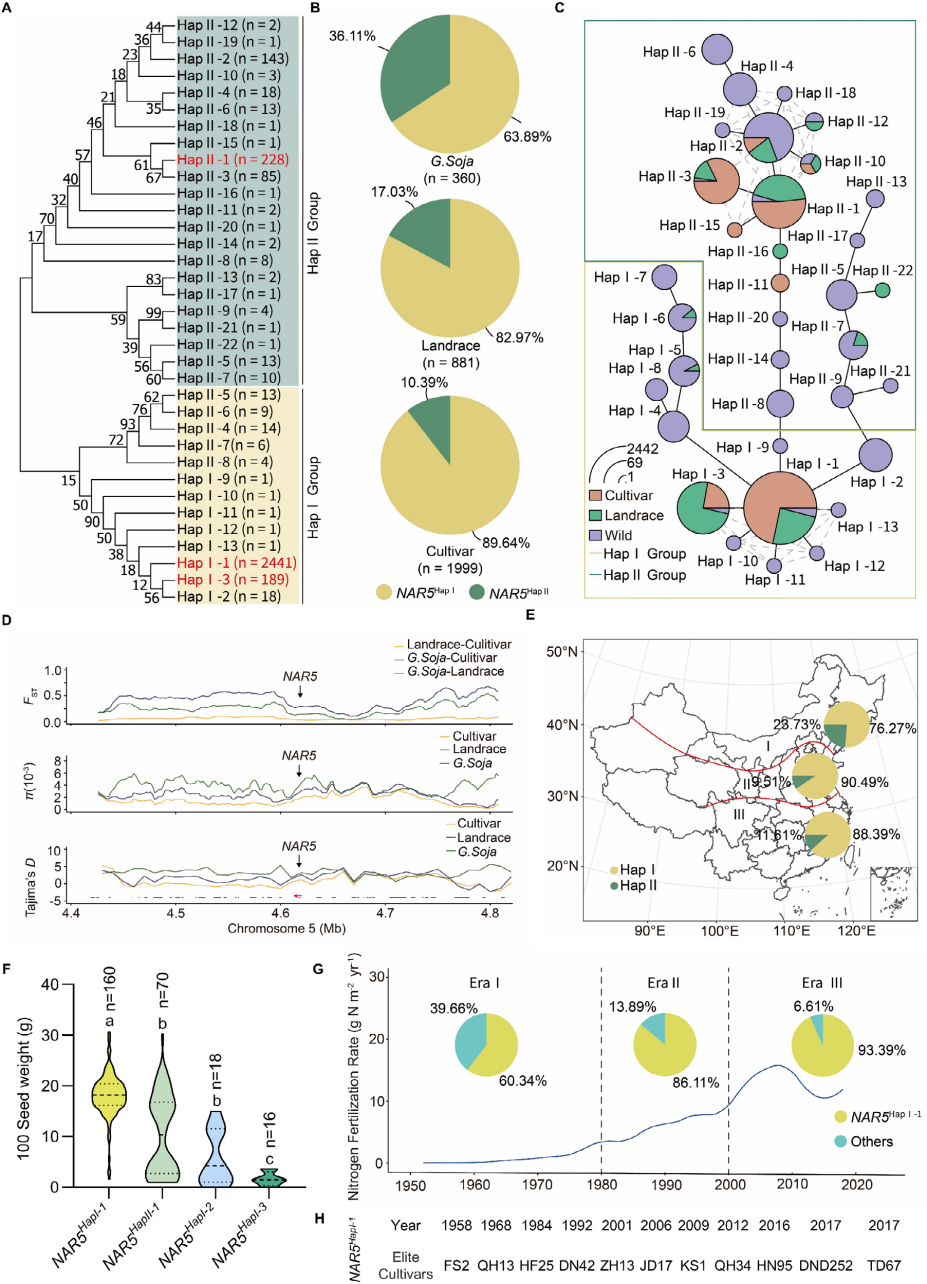
Genetic differentiation and selection of *NAR5* in soybean germplasm. (A) Phylogenetic analysis of *NAR5* haplotypes. (B) Haplotype‐frequency distribution for *NAR5* across 360 *G. soja* accessions, 881 *G. max* landraces, and 1999 improved cultivars. (C) *NAR5* haplotype network for *NAR5* ^HapI^ and *NAR5* ^HapII^. geneHapR was used to construct the haplotype network, with solid lines joining haplotypes with observed network links, which correspond to the most reliable and closely related evolutionary relationships. Inferred network connections are denoted by dashed lines, which correspond to potential but uncertain or indirect evolutionary relationships among haplotypes. (D) Selective sweep analysis around the *NAR5* locus. Panels (from top to bottom) depict patterns of: Population differentiation (*F*_ST_); Nucleotide diversity (*π*), and Tajima's *D* across wild soybeans, landraces and improved cultivars. Tajima's *D* values declined from 3.2408 in wild soybean to 2.6475 in landraces and 1.1840 in improved cultivars, and the Z‐score corresponding to the Tajima's *D* value of cultivar is 0.5127. A red dotted line depicts the location of the *NAR5* gene, while dark dotted lines indicate other genes. (E) Geographic distribution of the two major *NAR5* haplotype groups (*NAR5* ^HapI^ and *NAR5* ^HapII^) across China, including Ecoregion I (*n* = 1003 accessions), Ecoregion II (*n* = 768 accessions), and Ecoregion III (n = 620 accessions). (F) Association between *NAR5* haplotypes and seed weight. The 100‐grain weight of accessions harboring the *NAR5* ^HapI−1^ (*n* = 160 accessions), *NAR5* ^HapII−1^ (*n* = 70 accessions), *NAR5* ^HapI−2^ (*n* = 18 accessions), and *NAR5* ^HapI−3^ (*n* = 16 accessions) haplotypes are presented. (G) *NAR5* haplotype distributions in cultivated soybean and historical nitrogen application ratios. The Y‐axis corresponds to the nitrogen application ratio, derived from (Yu et al., *Earth Syst. Sci. Data*. 2022). Frequencies of *NAR5* ^HapI−1^ and other haplotypes are presented across Era I (*n* = 63 accessions), Era II (*n* = 220 accessions), and Era III (*n* = 626 accessions). (H) Elite cultivars harboring the *NAR5* ^HapI−1^ haplotype. Representative elite soybean cultivars carrying the *NAR5* ^HapI−1^ haplotype released since 1949 are presented.

Cultivated soybean (*Glycine max*) was derived from its wild progenitor (*Glycine soja* Siebold & Zucc.) approximately 5000–6000 years ago in China [44]. Genes governing SNF strongly influence yield, seed nutrient composition, and adaptability to varying soil nitrogen levels, making them potential targets of unconscious selection during domestication. To determine whether *NAR5* was subject to selection pressure, we analyzed sequence variation across 3240 soybean accessions, including 360 wild, 881 landrace, and 1999 improved cultivars [45]. This survey identified 35 haplotypes that clustered into two major groups: *NAR5* ^HapI^ and *NAR5* ^HapII^ (Figure 5A, Table S6). The frequency of *NAR5^HapI^* increased markedly from wild accessions to domesticated cultivars (Figure 5B). Among these, three dominant haplotypes (frequency >5%) were identified: *NAR5* ^HapI‐1^, *NAR5* ^HapI‐3,^ and *NAR5* ^HapII‐1^. Haplotype network analysis revealed that while *NAR5* arose from multiple wild ancestral lineages, *NAR5* ^HapI‐1^, which is a haplotype associated with enhanced nitrogenase activity compared with *NAR5* ^HapII^, became the predominant variant retained through domestication (Figure 5C). The results of nucleotide diversity (*π*) and population differentiation (*F*_ST_) indicate pronounced genetic differentiation at the *NAR5* locus between wild and cultivated soybean populations, suggesting that this region may have been subject to selection‐related pressures. However, Tajima's *D* did not exhibit a significantly negative value, indicating that this region did not undergo a typical recent strong selective sweep. Collectively, these findings suggest that NAR5 may have experienced domestication‐ or improvement‐related selection pressure during soybean domestication during the evolutionary transition from wild to cultivated soybean.

Given that nitrogen levels regulate symbiotic nitrogen fixation and nodule senescence, we hypothesized that natural genetic variation in *NAR5* correlates with topsoil nitrogen levels across different soybean‐growing ecoregions. To test this, we analyzed the geographic distribution of *NAR5* haplotypes in the sequenced soybean population. Overall, *NAR5* ^HapII^ occurred predominantly in Northeast China (Ecoregion I) and was less frequent in the Huang‐Huai‐Hai region (Ecoregion II), reflecting regional gradients in topsoil nitrogen concentration (Figure 5E, Figure S18A). This distribution pattern was largely driven by wild soybean accessions (Figure S18B). In contrast, the frequency ratio of *NAR5* ^HapI^ to *NAR5* ^HapII^ among landraces and cultivated lines progressively increased from higher to lower latitudes (Figure S18B,C). These spatial differences suggest that elevated soil nitrogen levels in northern regions may have relaxed selective pressure on *NAR5* in wild soybean. During domestication and subsequent breeding, the enrichment of *NAR5* ^HapI^ likely improved the adaptability of landraces and modern cultivars to a wider range of soil nitrogen conditions.

To further evaluate the agricultural relevance of *NAR5* allelic variants, we assessed 264 soybean accessions carrying either *NAR5* ^HapIs^ or *NAR5* ^HapII^. Since overexpression of *NAR5* enhances seed size and 100‐seed weight (Figure S19A–C), we specifically compared these traits between haplotypes and found that *NAR5* ^HapI‐1^ was associated with a significantly higher seed weight (Figure 5F). Tracking *NAR5* ^HapI‐1^ frequency across breeding eras (pre‐1980, 1980–2000, and post‐2000) revealed a steady increase from approximately 60% before 1980 to nearly 95% after 2000 (Figure 5G). Remarkably, this upward trend persisted despite substantial increases in nitrogen fertilizer use, indicating that elevated soil nitrogen did not reduce the prevalence or agronomic value of *NAR5* ^HapI‐1^ in elite cultivars (Figure 5G). Since the 1950s, many elite Chinese cultivars including Dongnong 42 (DN42), Zhonghuang 13 (ZH13), Dongongdou 252 (DND252), and Tiedou 47 (TD47), all have carried the *NAR5* ^HapI‐1^ genotype (Figure 5H). Collectively, these findings demonstrate that *NAR5* ^HapI‐1^, a haplotype enhancing both nitrogenase activity and seed weight, has undergone strong positive selection during modern soybean breeding.

### 2.8 Overexpression of *NAR5* Enhances Soybean Yield and Low‐Nitrogen Adaptability

**FIGURE 6:**
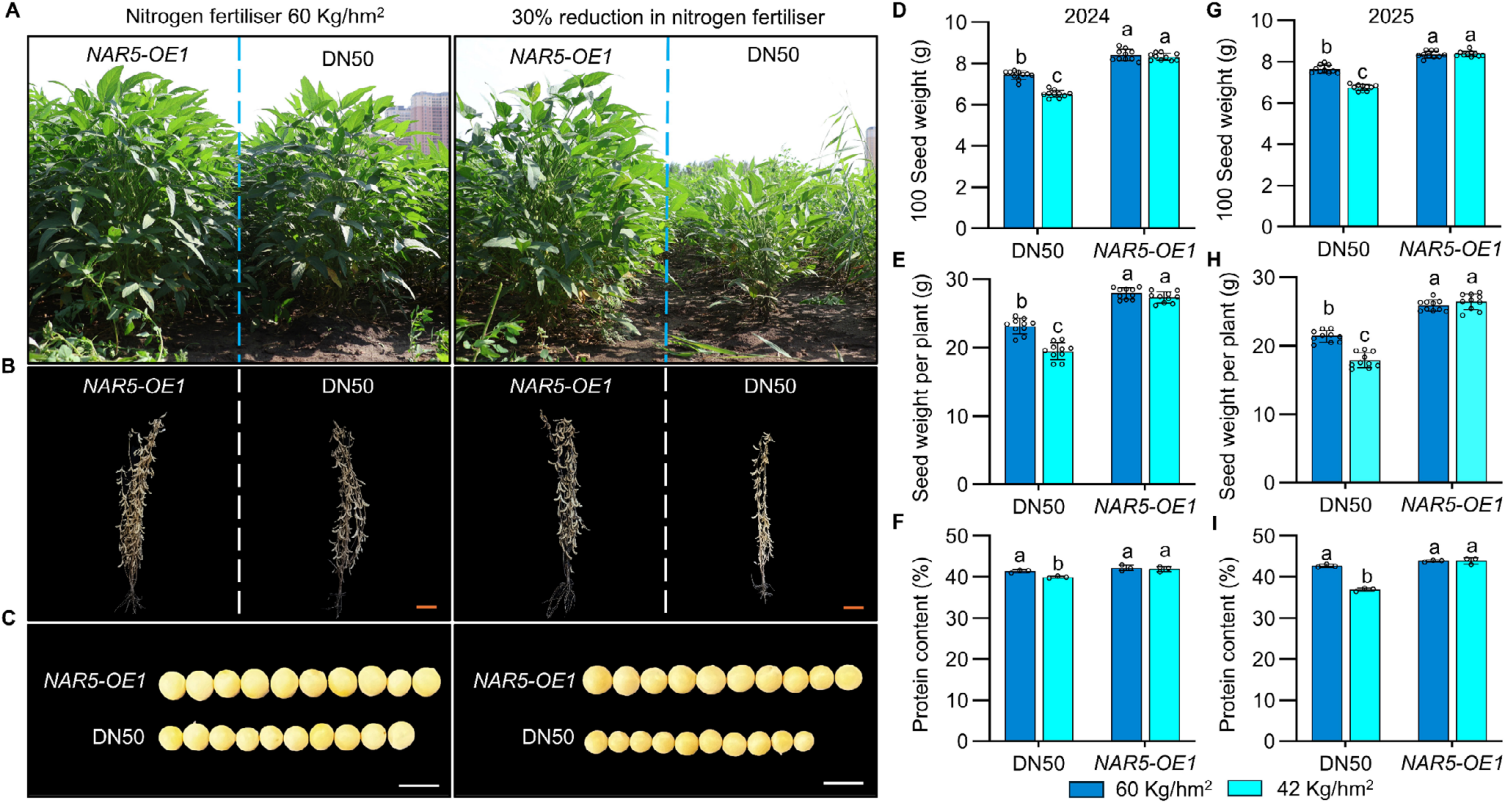
*NAR5* overexpression enhances soybean agronomic performance and adaptability to low‐nitrogen conditions. (A,B) *NAR5‐OE1* and DN50 plant phenotypes in the vegetative (A) and mature (B) stages, under standard (60 kg N ha^−^^1^) or low‐nitrogen (30% less, 42 kg N ha^−^^1^) treatments. Images in (A) were taken in August 2024; scale bars in (B) = 10 cm. (C) Quantification of seed widths in (B). (D–I) Data in (D–F) correspond to the 2024 field season, while data in (G–I) correspond to the 2025 field season.100‐seed weights (D,G), seed weight per plant (E, H), and seed protein content (F,I) for *NAR5‐OE1* and DN50 with nitrogen application at 60 kg N ha^−^^1^ of nitrogen fertilizer or reduced by 30% of nitrogen fertilizer application across the 2024 and 2025 growing seasons. Data are means ± SD (*n* = 10 biological replicates in D, E, G, and H; n = 3 biological replicates in F and I), and were compared using two‐way ANOVAs with Tukey's multiple‐comparison test (*p* < 0.05), and different lowercase letters indicate significant differences among groups.

To assess the potential of *NAR5* for the improvement of yield and nitrogen‐use efficiency in breeding programs, we compared agronomic performance between *NAR5‐OE1* and wild‐type (DN50) plants under field conditions during the 2024 and 2025 growing seasons. The plants were grown with a 30% reduction in nitrogen fertilizer without seed inoculation and reliance on indigenous rhizobia. Under nitrogen‐limited conditions, *NAR5‐OE1* plants exhibited visibly greater early growth vigor and canopy coverage compared with the wild type (Figure 6A,B). In 2024, preliminary small‐scale field trials showed that *NAR5* overexpression significantly increased seed length, 100‐seed weight and total yield per plant compared with DN50 (Figure 6D–F). Large‐scale field experiments in 2025 further demonstrated that *NAR5* overexpression effectively enhanced seed length, 100‐seed weight, total yield per plant and plot yield (Figure 6G–I, Figure S20). Plot yield increased by 12.65% under normal nitrogen fertilization, with a more pronounced improvement under 30% nitrogen reduction (Figure S20). Notably, wild‐type DN50 plants experienced substantial declines in both yield and protein levels under reduced nitrogen, whereas *NAR5‐OE1* maintained stable performance with no significant differences between reduced‐ and normal‐nitrogen conditions in 2024 and 2025 growing seasons (Figure 6C–G, Figure S20). These results confirm that *NAR5* overexpression boosts soybean productivity and confers resilience under nitrogen‐limited environments, effectively reducing dependence on synthetic nitrogen fertilizers.

## 3 Discussion

Soybean is the most important global source of plant‐derived oil and protein, prized for its high nutritional and industrial value. The rapidly growing global population and rising demand for protein‐rich foods have intensified the need to increase soybean yield [2, 46]. Unlike staple crops such as rice, wheat and maize, soybean engages in symbiotic nitrogen fixation (SNF), a trait critical for enhancing both yield and quality. However, only a few genetic loci directly regulating nodule nitrogenase activity have been identified [44]. Understanding the molecular mechanisms governing this process is therefore critical for advancing yield improvement. Through GWAS and QTL mapping under controlled greenhouse conditions, we identified *NAR5*, a gene encoding a subtilisin‐like serine protease that acts as a positive regulator of nodule nitrogenase activity. Consistent with breeding goals focused on enhanced SNF efficiency, *NAR5* overexpression substantially increased soybean yield under field conditions, particularly when nitrogen fertilizer inputs were reduced. These findings establish *NAR5* as both a functional determinant of symbiotic efficiency and a valuable genetic resource for developing high‐yield, low‐input soybean cultivars.

Plant SBTs form a large family of serine proteases involved in diverse physiological processes, including development, organogenesis, senescence, and defense signaling [31, 34]. Although transcriptomic analyses have revealed differential expression of certain *SBT* genes during plant–microbe interactions [47, 48, 49], their specific roles in symbiotic nitrogen fixation remain poorly characterized. Early work identified *Ag12* as the first *SBT* implicated in nodulation in *Alnus glutinosa*, where it was strongly expressed in the pre‐fixation zone of actinorhizal nodules [37]. Its homolog *Cg12* in *Casuarina glauca* is similarly induced during early infection but declines once nitrogen fixation is established [38]. In contrast, *NAR5* exhibits predominant expression in infected nodule cells, suggesting that individual SBT members may act on distinct substrates or at specific developmental stages to fine‐tune symbiotic processes. Comprehensive functional analysis of the *SBT* family will therefore be instrumental in uncovering the regulatory networks that govern nodule development and maintenance. Interestingly, NAR5 is relatively conserved within the nitrogen‐fixing clade, showing higher sequence similarity among leguminous species than with its *Arabidopsis* homolog (42.51% identity, Figure S3). This evolutionary conservation implies that NAR5 may have acquired specialized functions associated with symbiotic nitrogen fixation in legumes. Investigating the evolution and diversification of nodule‐expressed *SBT* genes across the nitrogen‐fixing clade will provide deeper insights into their specialized contributions to SNF.

SNF, while beneficial for nitrogen acquisition, requires substantial carbon investment from the host, as photosynthates are redirected to support rhizobial metabolism. Consequently, as plants shift resources toward reproductive growth, nitrogenase activity typically declines and nodules undergo senescence. Both premature and delayed senescence can negatively impact yield: insufficient nitrogen fixation limits growth and seed filling, whereas excessive fixation diverts carbohydrates away from developing pods. Optimizing the balance between carbon and nitrogen allocation (C–N economy) is therefore essential for achieving higher yield potential [50]. Our findings demonstrate that *NAR5* enhances nitrogenase activity while suppressing premature nodule senescence by downregulating senescence‐associated regulators such as *GmCYP35*, *GmNAC018*, and *GmNAC039* within nodule cells. This dual regulatory role positions *NAR5* as a key modulator of the C–N balance, enabling efficient nitrogen fixation without compromising reproductive yield. As a proteolytic enzyme with likely high substrate specificity or highly specific processing proteases, *NAR5* may act through the selective processing of target proteins that mediate nodule development and stress adaptation. Identifying these substrates will be crucial for elucidating the precise molecular mechanisms underlying its regulatory function and for expanding the genetic toolkit available for precision soybean breeding. Interestingly, *NAR5* exhibits a distinct spatial expression pattern from that of *GmNAC018* and *GmNAC039* and indirectly suppresses their expression. Although the precise molecular mechanism underlying this regulation remains unclear, future studies should aim to elucidate how *NAR5*, expressed in infected cells, may modulate intercellular signaling to influence the expression of *GmNAC018* and *GmNAC039*, which is also critical for optimizing soybean nitrogen fixation efficiency based on carbon‑nitrogen balance considerations.

Soybean was initially domesticated in China and subsequently spread worldwide [44, 51]. During this dispersal, selection for enhanced symbiotic efficiency has occurred, which proved crucial for its adaptation to diverse soil conditions and yield improvement. Even today, improving symbiotic nitrogen utilization remains a central objective in modern soybean breeding. However, while most domestication studies have emphasized yield‐ and quality‐related traits, comparatively little attention has been given to belowground traits, such as the genetic regulation of symbiotic nitrogen fixation. Selective sweep and haplotype analyses revealed that domestication favored the retention of *NAR5* haplotypes conferring higher nitrogenase activity, emphasizing that symbiotic efficiency has been an implicit breeding target. Over the past century, the prevalence of the elite haplotype *NAR5* ^HapI‐1^ increased dramatically, particularly after the 1980s, coinciding with continuous gains in 100‐seed weight and yield. From 1980 to 2000, breeding programs consistently selected for alleles linked to enhanced seed size and yield potential [51]. The enrichment of *NAR5* ^HapI‐1^, which promotes both nitrogenase activity and seed weight, likely contributed to the steady post‐1980 rise in seed mass. After 2000, high‐yield cultivars carrying *NAR5* ^HapI‐1^ became widely utilized as breeding parents, reinforcing the positive selection and widespread maintenance of this advantageous haplotype. Although nitrogen fertilization is typically known to suppress symbiotic nitrogen fixation, the adoption of *NAR5* ^HapI‐1^ remained strong even with increased fertilizer input. This persistence may indicate that *NAR5* ^HapI‐1^ is insensitive to nitrogen repression or that its yield‐promoting effect compensates for nitrogen‐mediated inhibition, which is an intriguing possibility deserving further study. Among the polymorphisms differentiating *NAR5* ^HapI‐1^ from other haplotypes, the causal variant responsible for differential promoter activity remains unresolved. Future exploration of upstream regulatory genes may reveal key promoter‐associated polymorphisms governing *NAR5* transcription, completing the broader regulatory network and potentially uncovering additional factors influencing nitrogenase activity. Such insights would provide valuable targets for precision breeding to enhance soybean performance.

In summary, these findings establish *NAR5* as a positive regulator of nitrogenase activity within infected nodule cells that simultaneously suppresses the expression of senescence‐associated genes in root nodules. The elite haplotype *NAR5* ^HapI‐1^, which enhances both nitrogenase activity and 100‐seed weight, has undergone strong selection throughout domestication and modern breeding. Moreover, *NAR5* overexpression confers improved yield potential and adaptability under low‐nitrogen conditions. Collectively, these results advance our understanding of the genetic mechanisms underlying nitrogen fixation efficiency in soybean nodules and offer a foundation for molecular design and breeding of high‐yield soybean cultivars.

## 4 Materials and Methods

### 4.1 Plant Materials, Rhizobia Inoculation, and Growth Conditions

A total of 309 soybean accessions were used in this study, including 96 wild soybeans (*Glycine soja*), 90 landraces, and 123 modern cultivars of *G. max*, as well as a recombinant inbred line (RIL) population (*n* = 147) derived from the cross Dongnong 594 × Charleston [52]. All soybean accessions were grown in sterile plant pots and arranged in a completely randomized design in the greenhouse, ensuring that all plants were maintained under identical environmental conditions (200 µmol photons m^−2^ s^−1^, 16‐h light/8‐h dark, 25°C). All accessions were inoculated with *Sinorhizobium fredii* HH103 (“HH103”) for nitrogenase activity assays. Four weeks after inoculation with HH103, five representative plants were selected from each accession and their individual nitrogenase activities were measured. The mean activity value per accession was then used for subsequent Genome‐Wide Association Study (GWAS) and quantitative trait locus (QTL) mapping.

The *G. max* cultivar Dongnong 50 (DN50) cultivar was used when generating stable transgenic *NAR5_pro_: NAR5–GFP* lines (*NAR5‐OE1* and *NAR5‐OE2*), *nar5* mutants (*nar5‐1* and *nar5‐2*), and stable‐transgenic *NAR5‐*silenced lines (*NAR5‐Ri1* and *NAR5‐Ri2*).

HH103 and HH103 *nifH:GUS* were grown at 28°C in TY medium. For HH103 *nifH:GUS*, was generated by cloning the *nifH* promoter and *β‐glucuronidase* (*GUS*) coding sequence into the pFAJ1702 vector, followed by introduction into HH103 via triparental mating.

For nitrogenase assays, soybean seeds were surface‐sterilized using chlorine gas for 12 h and germinated in vermiculite under controlled greenhouse conditions (200 µmol photons m^−2^ s^−1^, 16‐h light/8‐h dark, 25°C). Seedlings were supplied with a 1 mM nitrogen nutrient solution. Nitrogenase activity in nodules was quantified at four weeks post‐inoculation (4 wpi) with HH103. Soybean nodules from all accessions or mutant lines were sampled and assayed for nitrogenase activity in a random order to minimize batch effects during sample processing.

*Nicotiana benthamiana* seedlings were cultivated in a greenhouse at 25°C under a 16‐h light/8‐h dark photoperiod with 150 µmol photons m^−2^ s^−1^ illumination. Four‐week‐old plants were infiltrated with *Agrobacterium tumefaciens* GV3101 to assess the subcellular localization and promoter activity of *NAR5* ^HapI^ and *NAR5* ^HapII^.

### 4.2 Soybean Stable Transformation and Gene Editing

Stable transformation of soybean was performed following established protocols [53, 54]. To construct *NAR5* overexpression lines, the 2.5‐kb promoter region upstream of the translation start site and the *NAR5* coding sequence from DN50 (*NAR5* ^HapI^) were fused with *GFP* and inserted into the pTF101 vector containing the Bialaphos Resistance (*BAR*) gene as a selectable marker. The resulting plasmids were introduced into *A. tumefaciens* EHA105 and transformed into DN50 cotyledonary nodes. Overexpression efficiency was confirmed by immunoblotting. Stable *NAR5* silencing lines (*NAR5‐Ri1* and *NAR5‐Ri2*) were generated using the same method.

For the creation of the *nar5‐1* and *nar5‐2* mutants, CRISPR–Cas9 gene editing was employed. A guide RNA (CCTCGTACCACAAGCAGTTCAAA) was designed using CRISPR‐P 2.0 (http://crispr.hzau.edu.cn/CRISPR2/and cloned into pGES401 via Golden Gate assembly [55]. The construct was transferred into *A. tumefaciens* EHA105 and transformed into DN50 cotyledonary nodes. Genomic DNA from regenerated plants was amplified with gene‐specific primers, and PCR products were sequenced to confirm target editing.

### 4.3 Soybean Hairy‐Root Transformation

To analyze *NAR5* expression patterns and promoter‐driven transcriptional differences among haplotypes (*NAR5* ^HapI‐1,2 and 3^ and *NAR5* ^HapII^), the *NAR5^Haps^_pro_:GUS* reporter constructs were cloned into pTF101 and introduced into *Agrobacterium rhizogenes* K599 for soybean hairy‐root transformation, as previously described [53, 56]. Briefly, hypocotyls from germinating DN50 seedlings were excised and incubated for 1 h in LCCM medium (1/10× Gamborg B5 basal salts, 88 mM sucrose, 20 mM MES, and 0.2 mM acetosyringone; pH 5.4) containing *A. rhizogenes* K599 harboring *NAR5^Haps^_pro_:GUS*. After bacterial removal, seedlings were transferred to CCM medium (1/10× Gamborg B5 basal salts, 88 mM sucrose, 20 mM MES, 3.3 mM cysteine, 1 mM dithiothreitol, and 0.2 mM acetosyringone; pH 5.4) and cultured in darkness for 72 h at 25°C. The seedlings were then transferred to rooting medium (1/10× Gamborg B5 basal salts, 88 mM sucrose, 20 mM MES, 7.5 g·L^−^^1^ agar; pH 5.4).

To evaluate how natural variation in the *NAR5* coding sequence influences nitrogenase activity, the *NAR5* promoter from DN50 (*NAR5* ^HapI^) and the coding sequence of *NAR5* ^HapI^ or *NAR5* ^HapII^ were fused with GFP, cloned into pTF101, and introduced into *A. rhizogenes* K599. A control transformation using *NAR5^DN50^_pro_:GFP* was conducted in parallel. Nitrogenase activity of the resulting root nodules was measured at 4 wpi following HH103 inoculation.

To further assess the functional impact of *NAR5* mutations, *NAR5–GFP* driven by the DN50 *NAR5* promoter was expressed in both *nar5‐1* mutants and DN50 wild‐type backgrounds via hairy‐root transformation. *A. rhizogenes* K599 carrying *NAR5^DN50^_pro_:GFP* transformed into the *nar5‐1* background served as the negative control.

### 4.4 Nitrogenase Activity Assay

Nitrogenase activity was quantified using a modified version of the acetylene reduction assay (ARA) as described in established protocols [4]. In brief, soybean root nodules of uniform size were harvested and sealed in 15 mL glass vials. Two milliliters of air were withdrawn from each vial and replaced with an equal volume of acetylene gas. The sealed vials were incubated at room temperature for 1 h to allow the enzymatic conversion of acetylene to ethylene. The reaction was then terminated by injecting 1 mL of 1 mol L^−1^ NaOH into each vial, which was subsequently resealed. A 1 mL gas sample from each reaction vial was analyzed for ethylene content using a gas chromatograph (GC‐14, Japan). Nitrogenase activity was expressed relative to the fresh weight of nodules and/or on a per‐plant basis.

### 4.5 GWAS and QTL Mapping for the Root Nodule Nitrogenase Activity Trait

For GWAS, a dataset containing 6 987 829 high‐confidence single nucleotide polymorphisms (SNPs) from whole‐genome resequencing of 309 soybean accessions was used to evaluate genetic loci associated with nodule nitrogenase activity. Population structure was characterized using the Bayesian clustering algorithm implemented in fastStructure. SNPs with a minor allele frequency (MAF) ≤0.05 or a missing data rate ≥0.1 were excluded. Association analyses were performed using a mixed linear model (MLM) in the Efficient Mixed‐Model Association expedited (EMMAX) software package. A pairwise kinship matrix was calculated based on the simple matching coefficient from the variance‐covariance matrix of random effects to account for relatedness among accessions.

A permutation‐based test was applied to establish the genome‐wide significance threshold [1, 57]. In this procedure, phenotypic data were randomly permuted to disrupt genotype–phenotype associations, and the GWAS was re‐run using the same model. The smallest *p*‐value observed across the genome in each of 1000 permutations was recorded. The 95th percentile of this distribution was used as the genome‐wide significance threshold, corresponding to a type I error rate of 5%. PopLDdecay to estimate linkage disequilibrium (LD) decay across 309 soybean accessions, and the distance of LD decayed to half of its maximum value was defined as the LD decay distance. Using this metric, we delineated candidate intervals by extending one LD decay distance in both directions from each SNP that reached the genome‐wide significance threshold. The local LD boundaries defined by *r* ^2^ > 0.2 using LDBlockShow (v1.40) [58, 59].

For QTL mapping, 147 recombinant inbred lines (RILs) derived from a cross between the cultivars Charleston and Dongnong 594 (DN594) were advanced to the F_10_ generation via single‐seed descent [52, 60]. QTL analysis was performed using QTL IciMapping (v4.2), and the significance threshold was determined through 1000 permutations at *p* < 0.05. For the RILs population, the BIP (biparental population) module was applied, and QTL mapping was conducted using the inclusive composite interval mapping (ICIM) method. Confidence interval calculated by 1‐LOD drop from the estimated QTL position.

### 4.6 Phylogenetic Analysis

Homologs of the GmNAR5 protein were identified through BLAST searches using Phytozome 14 (https://phytozome‐next.jgi.doe.gov/blast‐search). Genes sharing greater than 40% sequence identity in *Arabidopsis thaliana* and more than 60% identity in *Glycine max* and other representative species including *Arabidopsis thaliana*, *Zea mays*, *Oryza sativa*, *Medicago truncatula*, *Phaseolus vulgaris*, *Cicer arietinum*, *Lotus japonicu*s, *Vigna angularis*, *Vigna unguiculata*, *Lupinus albus*, *Lupinus angustifolius*, *Aeschynomene evenia*, *Arachis hypogaea*, *Mimosa bimucronata*, *Prosopis alba*, *Ziziphus jujuba*, *Morus notabilis*, *Prunus persica*, *Juglans regia*, *Alnus glutinosa*, *Parasponia andersonii* and *Pisum sativum* were included. Multiple sequence alignments and phylogenetic trees were generated using the neighbor‐joining method implemented in MEGA 11.0, with 1000 bootstrap replications for statistical support. The resulting tree was visualized and refined using iTOL (https://itol.embl.de/).

### 4.7 GUS Staining and Activity Assay

Histochemical β‐glucuronidase (GUS) staining was conducted on soybean root nodules to characterize promoter activity and spatial–temporal expression patterns of various *NAR5* haplotypes. Nodules collected at four weeks post‐inoculation (4 wpi) were immersed in GUS staining solution (2 mM X‐Gluc, 50 mM NaH_2_PO_4_, 50 mM Na_2_HPO_4_, 0.5 mM K_3_Fe(CN)_6_, 0.5 mM K_4_Fe(CN)_6_, and 0.2% Triton X‐100, pH 7.2). Samples were vacuum‐infiltrated for 20 min and incubated overnight at 37°C in the dark. GUS‐stained nodules were visualized under a stereomicroscope (Leica MZ10F). The nitrogenase activity was characterized using HH103 *nifH_pro_:GUS*, following a previously described method.

Quantitative GUS enzymatic activity was measured fluorometrically using 4‐methylumbelliferyl‐β‐D‐glucuronide (4‐MUG) as substrate [61, 62]. Root nodules were homogenized in phosphate‐buffered saline (PBS, pH 7.0) supplemented with β‐mercaptoethanol and detergent. After centrifugation, 500 µL of the supernatant was incubated with 1 mM 4‐MUG at 37°C for 10 min, and the reaction was terminated by adding 0.2 M sodium carbonate. The resulting fluorescence of 4‐methylumbelliferone (4‐MU) was quantified with a spectrofluorometer at 365 nm excitation and 455 nm emission wavelengths. Concentrations were determined from a 4‐MU standard curve, and enzyme activity was expressed as picomoles of 4‐MU released per minute per milligram of protein. Protein concentrations were measured using the Bradford assay (Detergent‐Compatible Bradford Kit, Sangon C503081) for normalization.

### 4.8 In Situ Hybridization

RNA in situ hybridization followed previously established procedures [63]. Soybean nodule tissues harvested at 2, 4, 6, 8, and 10 weeks post‐inoculation were fixed at 4°C in an RNase‐free solution containing 3.7% formaldehyde, 50% ethanol, and 5% acetic acid. A 130‐bp fragment of *NAR5* cDNA containing T7 and SP6 promoter sequences was amplified and inserted into the pSPT18 T‐Easy vector (Roche, 11175025910). Antisense and sense RNA probes were synthesized using the DIG RNA Labeling Kit (Roche, 11175025910) following the manufacturer's instructions. Hybridization signals were visualized using 3,3′‐diaminobenzidine (DAB) staining and imaged under a Leica DVM6 stereomicroscope.

### 4.9 Physiological Observations

To assess internal coloration, soybean nodules were bisected longitudinally with a razor blade and immediately examined under a Leica MZ10F stereomicroscope using identical imaging conditions for all samples.

Leghemoglobin content was quantified following established methods [64, 65]. Approximately 1 g of freshly collected nodules was homogenized in 0.1 M phosphate buffer (PBS, pH 6.4) at 4°C. The homogenate was centrifuged at 12 000×*g* for 20 min, and the absorbance of the supernatant was measured at 520, 540, and 560 nm, with PBS serving as a blank control.

For TEM, nodule segments approximately 2 mm in length were fixed in 2.5% glutaraldehyde (Servicebio, G1102) under vacuum for 10 min and stored at 4°C. After fixation, samples were washed five times in 0.1 M PBS, post‐fixed with 1% osmium tetroxide for 4 h, and rinsed again. The samples were dehydrated through an ethanol series, embedded in EPON 812 resin, and sectioned into 70 nm ultrathin slices using a Leica UC7 ultramicrotome. Sections were examined using a FEI Tecnai G2 Spirit BioTWIN transmission electron microscope operating at 100 kV.

### 4.10 RNA‐Seq and Data Analysis

To assess transcriptional changes associated with *NAR5* overexpression, RNA‐seq profiling was performed on nodules from the transgenic line *NAR5‐OE1* and compared with those of the wild‐type cultivar DN50, both inoculated with wild‐type HH103. Nodule samples were collected at 4 and 8 weeks post‐inoculation (wpi). Total RNA was extracted using FreeZol Reagent (Vazyme, R711‐01). For each treatment, nodules from five individual plants were pooled as one biological replicate, and three independent replicates were analyzed per condition.

RNA‐seq libraries were constructed from these biological replicates and sequenced on the Illumina NovaSeq 6000 platform to generate paired‐end reads. Raw reads were processed using fastp (v0.23.4) for adapter removal and quality filtering with default parameters. Clean reads were aligned to the *Glycine max* reference genome (Wm82.a6.v1) using HISAT2 (v2.2.1) in strand‐specific mode. Transcript reconstruction followed a reference‐guided approach using StringTie, and transcript abundance was quantified in RSEM (v1.3.3) as transcripts per million (TPM). DEGs were identified using DESeq2 (v1.42.0). Genes were considered significantly differentially expressed when |log_2_(fold change)| ≥ 1 and false discovery rate (FDR) ≤ 0.05.

### 4.11 *NAR5* Haplotype Analysis

Sequence variation across the 3‐kb promoter and full‐length genomic region of NAR5 was examined using resequencing data from 3240 soybean accessions [45]. Variant filtering was carried out in VCFtools (v0.1.17), applying thresholds of minor allele frequency (MAF) > 5% and missing data < 10%. Nonfunctional variants and polymorphisms showing weak association signals were excluded, yielding a final dataset of 65 high‐confidence SNPs. Haplotype classification was performed in R using the geneHapR package (v1.2.5), which grouped accessions into three major haplotypes based on shared polymorphisms.

To explore evolutionary relationships among these haplotypes, a haplotype network was generated in geneHapR using the functions get_hapNet and plotHapNet for network construction and visualization. The get_hapNet function utilizes pegas::haploNet to infer phylogenetic relationships among haplotypes.

### 4.12 Selection Analysis

Genetic differentiation (*F*_ST_), nucleotide diversity (π), and Tajima's *D* were calculated using VCFtools (v0.1.13). Analyses employed a 20‐kb sliding window with a 2‐kb step size. The upper 5% of genome‐wide values was used as the empirical threshold for identifying regions under selection.

### 4.13 Phenotyping of Agronomic Traits

For the field experiments, *NAR5* overexpression line (*NAR5‐OE1*) and wild‐type DN50 were planted under a randomized block design with five replicates of each genotype in experimental fields over the 2024 and 2025 growing seasons at the Transgenic Research Field of Northeast Agricultural University, Harbin, China (45°44′N, 126°43′E). In 2024, seeds were sown in early May in single rows 5 m in length, with 5 cm between plants and 60 cm between rows, achieving a planting density of approximately 300 000 plants ha^−1^. In 2025, the plots were designed as five 10 m‐long rows per plot (∼30 m^2^), with 5 cm between plants and 60 cm between rows. Soil available nitrogen content prior to basal fertilization of the field was 126.42±7.62 mg kg^−1^ in 2024 and 122.81 ± 3.48 mg kg^−1^ in 2025 (Table S7). In the experimental plots, urea application was reduced by 30% (42 kg N ha^−1^) relative to the control (60 kg N ha^−1^). All plots received a uniform application of compound fertilizer containing 18% N, 16% P_2_O_5_, and K_2_O (>48% total nutrients) at a rate of 150 kg ha^−1^. Throughout the 2024 and 2025 field trials, management was standardized such that no supplementary irrigation or inoculation with rhizobia was implemented. At physiological maturity in October, 10 randomly selected individual plants of DN50 or *NAR5‐OE1* were harvested in 2024 and 2025. Agronomic traits were measured, including 100‐seed weight, seed protein concentration, and yield per plant. Seed protein content was quantified using an NDA702 Dumas nitrogen analyzer (Velp Scientifica).

### 4.14 Statistical Analysis

All statistical analyses were conducted in GraphPad Prism (v10.4.0). Results represent data from a minimum of three biological replicates. For violin and box plots, the box boundaries indicate the interquartile range (25th–75th percentiles), with the median represented by a central horizontal line. Statistical significance was determined using Student's t‐test or Tukey's multiple comparison test, with *p* < 0.05 considered significant and *p* < 0.01 denoting high significance.

### 4.15 Accession Numbers

Accession numbers for this study are as follows: NAR5 (Glyma.05G051500), GmUNK1 (Glyma.12G020500), GmNAC018 (Glyma.04G208300), GmNAC039 (Glyma.06G157400) and GmLbC1 (Glyma.10G199000).

## Author Contributions

C. M., J.W., D.X. Q.S., and C.L. conceived the project and designed experiments. H.Z., H.F., L.D., L.C., M.Y., R.C., and J.C. performed experiments, conducted fieldwork, analyzed data. C.M. and J.W. wrote the article. X.W. and Q.S. revised the manuscript. All authors read and approved the final manuscript.

## Conflicts of Interest

The authors declare no conflicts of interest.

## Supporting information




**Supporting File 1**: advs75316‐sup‐0001‐SuppMat.docx.

**Supporting File 2**: advs75316‐sup‐0002‐TableS1.xlsx.

**Supporting File 3**: advs75316‐sup‐0003‐TableS2.xlsx.

**Supporting File 4**: advs75316‐sup‐0004‐TableS3.xlsx.

**Supporting File 5**: advs75316‐sup‐0005‐TableS4.xlsx.

**Supporting File 6**: advs75316‐sup‐0006‐TableS5.xlsx.

**Supporting File 7**: advs75316‐sup‐0007‐TableS6.xlsx.

**Supporting File 8**: advs75316‐sup‐0008‐TableS7.xlsx.

## Data Availability

All raw sequencing data from three replicates of RNA‐seq for each treatment in this study have been deposited in the China National Center for Bioinformation (https://ngdc.cncb.ac.cn/bioproject; PRJCA048078; submission subPRO070555 and subCRA051432). Raw sequencing data for the 309 soybean accessions have been deposited at the NCBI Sequence Read Archive (https://www.ncbi.nlm.nih.gov/sra; PRJNA910939, SUB12395141). The data that support the findings of this study are available from the corresponding author upon reasonable request.
