# Supplementary material for: Natural Variation of NAR5 Determines Nitrogenase Activity and the Yield in Soybean
Source: Adv Sci (Weinh). 2026 Apr 14;13(38):e21100. doi: 10.1002/advs.202521100 (PMC13335570; doi:10.1002/advs.202521100)
Supplement: Supplementary file 1 — Supporting File 1: advs75316‐sup‐0001‐SuppMat.docx. [file ADVS-13-e21100-s001.docx]

**Supplementary Information**

**Natural variation of *NAR5* determines nitrogenase activity and the yield in soybean**

Chao Ma^1, 2†^, Hanyu Zhao^1, 2†^, Haojie Feng^3†^, Xulun Dong^1†^, Lin Chen^1^, Mingliang Yang^1, 2^, Runyi Chen^1^, Chengjun Lei^1^, Chunyan Liu^1, 2*^, Qingshan Chen^1, 2*^, Dawei Xin^1, 2*^, Jinhui Wang^1, 2*^

^1^ National Key Laboratory of Smart Farm Technology and System, College of Agriculture, Northeast Agricultural University, Harbin 150030, P.R. China.

^2^ Key Laboratory of Soybean Biology in Chinese Ministry of Education, College of Agriculture, Northeast Agricultural University, Harbin 150030, P.R. China

^3^ Suihua Branch of Heilongjiang Academy of Agricultural Sciences, Suihua 152052, P.R. China.

^†^ Chao Ma, Hanyu Zhao, Haojie Feng and Xulun Dong contributed equally to this work.

^*^ Correspondence: Jinhui Wang, Email: jinhuiwang113@126.com;

Dawei Xin, Email: dwxin@neau.edu.cn;

Qingshan Chen, Email: [qshchen@126.com](mailto:qshchen@126.com);

Chunyan Liu, Email: cyliucn@neau.edu.cn.

**This supplementary information file contains:**

**Supplemental Figure 1 to 20**

**List of Supplemental Tables**

**Supplemental** **Figure Legends**

**Supplemental Figure 1.** **Nitrogenase activity levels in the RIL parents**

Nitrogenase activity levels in the RIL parents (Charleston and DN594). Data are means ± SD (n = 11 biological replicates), and were compared with Student’s *t***-**tests (**, *P* < 0.05).

**
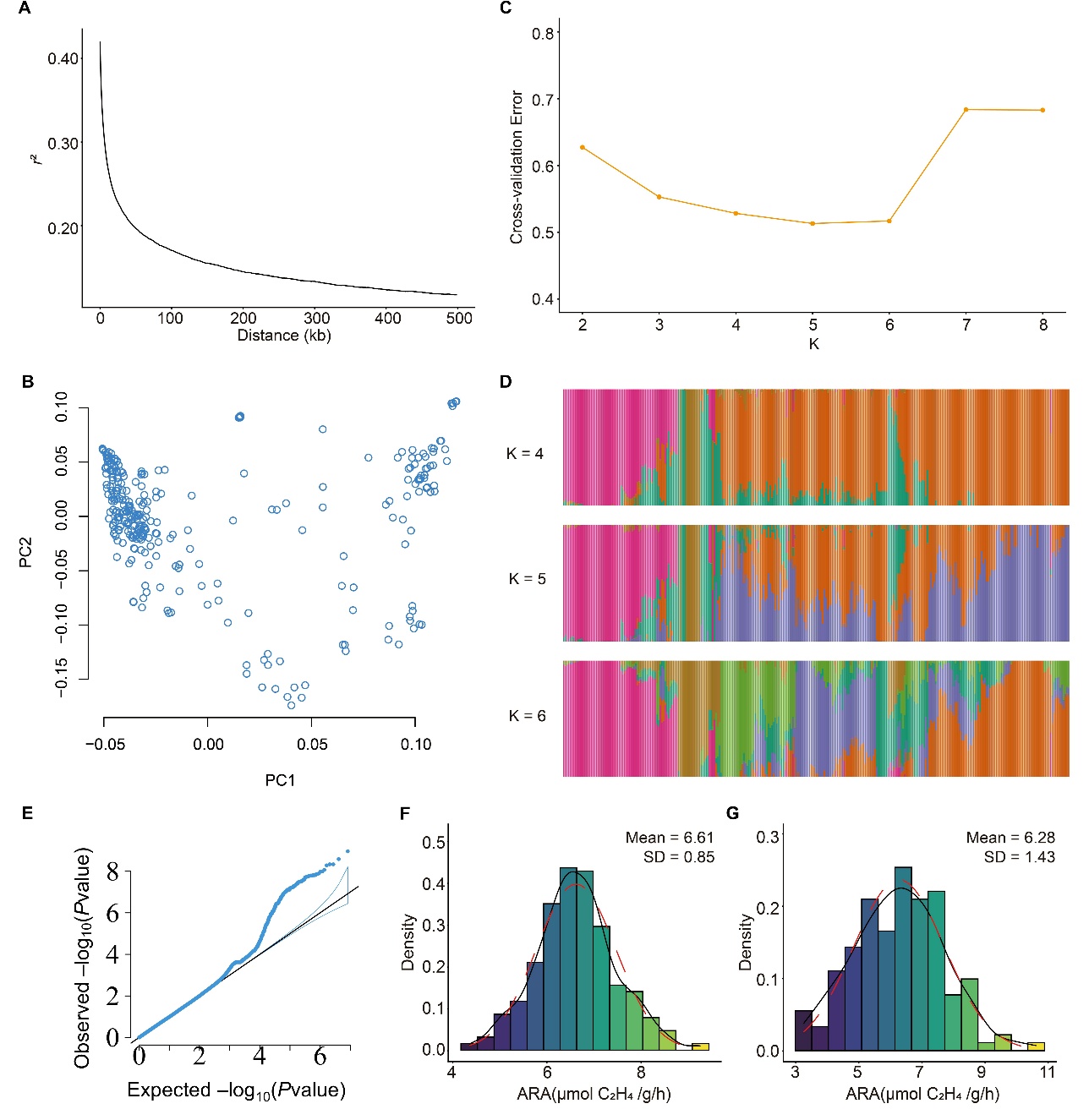
**

**Supplemental Figure 2.** **Population structure of 309 soybean accessions and nitrogenase activity quantile-quantile plot.**

**(A)** LD decay plot of 309 soybean accessions. The analysis was performed using PopLDdecay on the entire population. The LD decay distance was determined to be 19,994 bp, corresponding to the point where the r^2^ value (r^2^ = 0.2366) dropped to half of its maximum. This distance was used to define the flanking regions for identifying candidate regions around significant GWAS loci. **(B)** Principal component analysis (PCA) plot showing the genetic structure of 309 re‑sequenced soybean accessions, analyzed using PLINK (version 1.9). **(C)** Cross-validation error (CV error) plot derived from fastStructure analysis (K = 2–8). The line chart displays CV error values (y-axis) against different assumed numbers of genetic clusters, K (x-axis). The lowest CV error (0.51325) was observed at K = 5, while the values at K = 4 and K = 6 were 0.52841 and 0.51698, respectively. **(D)** Population structure of 309 soybean accessions estimated, displayed for K values of 4, 5 and 6. **(E)** A quantile-quantile plot plotting −log_10_-transformed observed *P*-values against −log_10_-transformed expected *P*-values. Genomic inflation factor (λ_CG_) = 1.5138. **(F)** Histogram of the nitrogenase activity distribution across 309 natural accessions. The black curve represents the kernel density estimation (KDE), and the red curve indicates the fitted normal distribution. The mean and standard deviation (SD) are annotated in **(F)**. **(G)** Histogram of the nitrogenase activity distribution across soybean recombinant inbred line (RIL, n = 147) population from DN594 × Charleston. The black curve represents the KDE, and the red curve indicates the fitted normal distribution. The mean and SD are annotated in **(G)**.


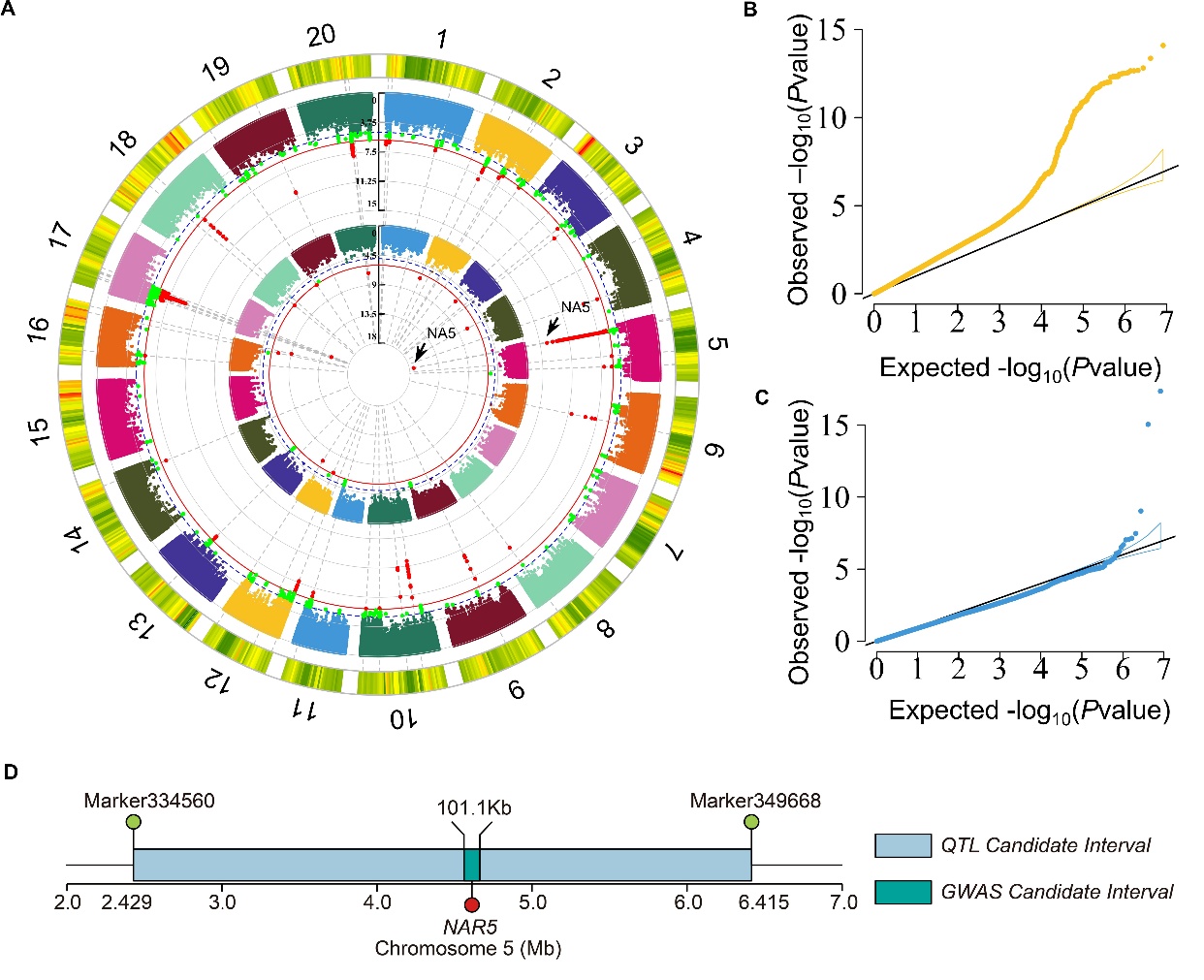


**Supplemental Figure 3. Nitrogenase activity GWAS analysis.**

**(A)** Manhattan plots from GLM (outer) and FarmCPU (inner) models. **(B, C)** Quantile-quantile plots depicting the observed versus expected *P*-value distributions for the GLM **(B)** and FarmCPU **(C)** models. The genomic inflation factor (λ_CG_) of FarmCPU was 1.0378. **(D)** The physical position and relative chromosomal location of the candidate interval. 101.1-kb interval the overlap between a QTL confidence interval and a local LD block around the GWAS lead SNP.


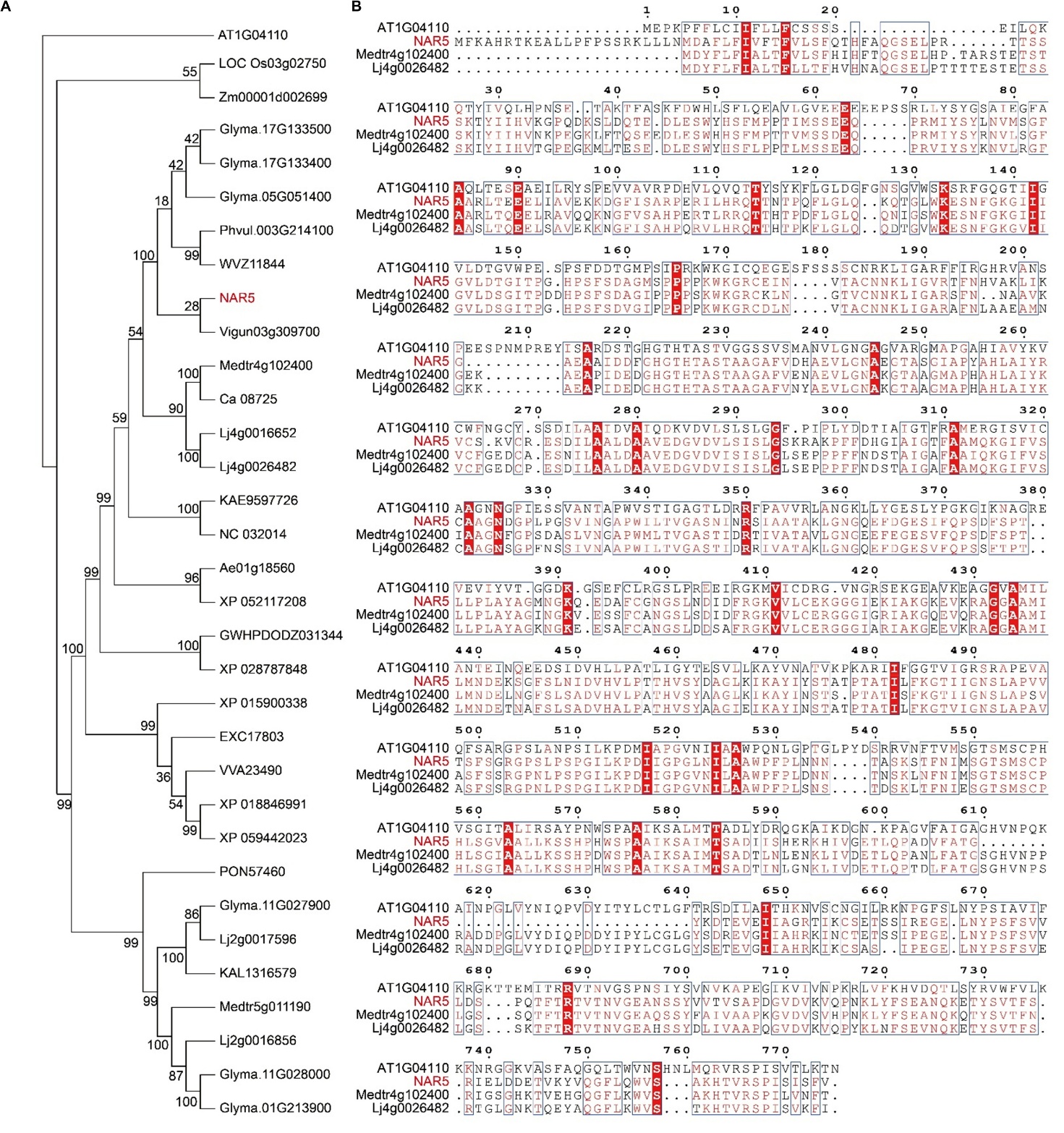


**Supplemental Figure 4. Phylogenetic analysis of NAR5 orthologs.**

**(**A) Neighbor-joining phylogenetic tree of select NAR5 orthologs. NAR5 ortholog and homolog alignment was performed with Clustal-W, analyzing sequences from *Glycine max*, *Arabidopsis thaliana*, *Medicago truncatula*, and *Lotus japonicus*. **(**B) NAR5 alignment with select homologs from *A. thaliana*, *M. truncatula, and L. japonicus*.


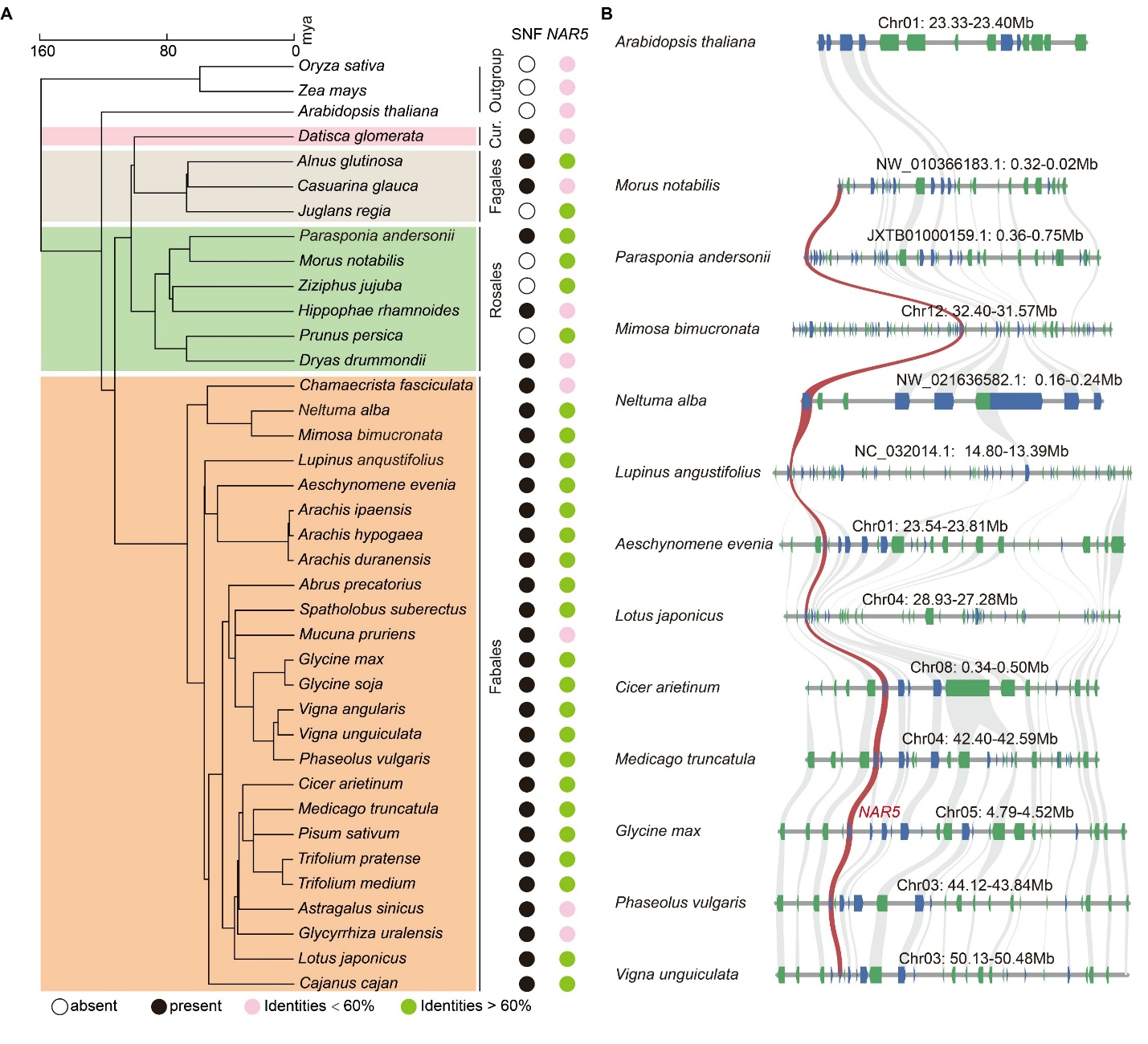


**Supplemental Figure 5.** **Phylogenetic and syntenic relationships among NAR5 homologs from NFC and non-NFC species.**

**(**A) Phylogenetic relationships among species within and outside the nitrogen-fixing clade (NFC), including *Fabales*, *Fagales*, *Cucurbitales* (*Cuc*.), and *Rosales*, as well as the non-NFC species *Arabidopsis thaliana*, *Zea mays*, and *Oryza sativa*. The circles on the right side of the tree denote the presence or absence of SNF and the degree of NAR5 homolog sequence similarity. The scale bar in the upper left represents the estimated divergence time in million years ago (mya). Findings are based on publicly available data (https://www.ncbi.nlm.nih.gov/). **(**B) Syntenic relationships between *Arabidopsis thaliana* and other NFC species. Blue and green rectangles denote genes within the syntenic blocks identified among species. Red lines highlight homologous genes corresponding to NAR5, while gray lines connect other syntenic but non-homologous genes.

**
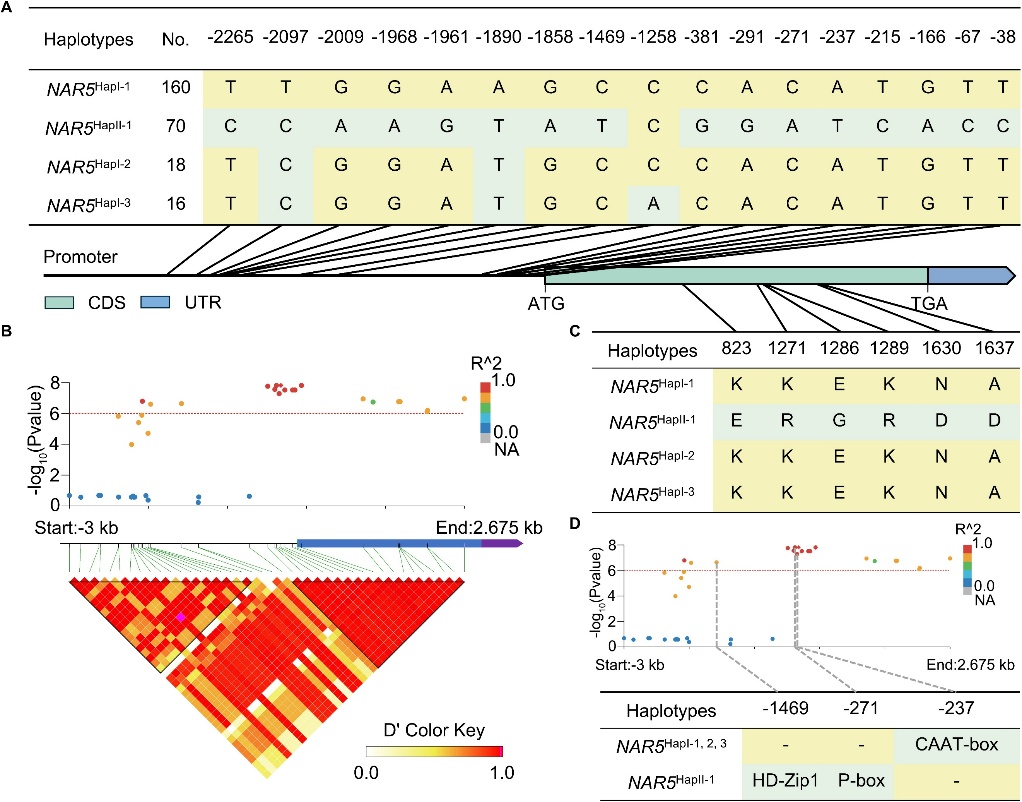
**

**Supplemental Figure 6. Haplotype analysis of sequence variation in the 3-kb NAR5 promoter region and full-length gene.**

**(**A) Natural variations in *NAR5*^HapI-1^, *NAR5*^HapII-1^, *NAR5*^HapI-2^, and *NAR5*^HapI-3^ promoters. **(**B) Genome-wide Manhattan plot (upper) and linkage disequilibrium plot (lower) in the 3-kb *NAR5* promoter region and full-length gene. The signal threshold is marked with a dashed line. The color gradient from white to red represents pairwise r^2^ values, corresponding to the degree of LD. **(**C) Natural variations in *NAR5*^HapI-1^, *NAR5*^HapII-1^, *NAR5*^HapI-2^, and *NAR5*^HapI-3^ for the full-length *NAR5* gene. **(**D) Natural variations in the *NAR5* promoter are associated with altered cis-regulatory elements.


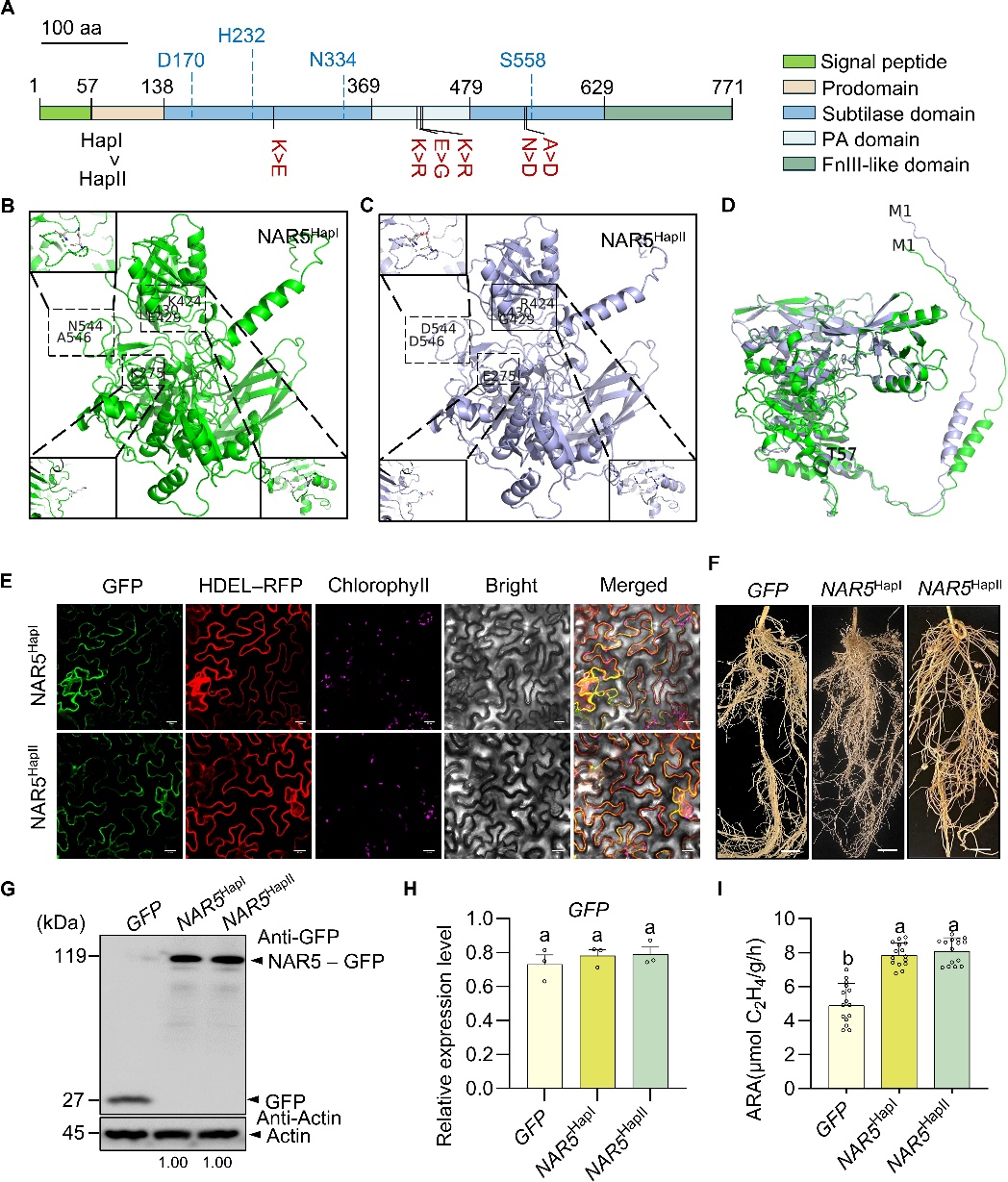


**Supplemental Figure 7. Coding sequence variation in *NAR5* does not alter nitrogenase activity levels.**

**(A)** *NAR5* gene structure and natural variant locations. Amino acid changes that define the HapI and HapII haplotypes were attributable to five SNPs in the coding sequence; blue shading indicates the conserved subtilase family active site. **(B, C)** *NAR5*^HapI^ **(B)** and *NAR5*^HapII^ **(C)** protein structures as predicted by AlphaFold3. **(D)** Structural alignment of *NAR5*^HapI^ and *NAR5*^HapII^. **(E)** *NAR5*^HapI^ and *NAR5*^HapII^ subcellular localization in tobacco leaves. The HDEL–RFP protein is an endoplasmic reticulum marker. Scale bars = 20 µm. **(F)** Nodule phenotypes from DN50 hairy roots expressing GFP, *NAR5*^HapI^-GFP, or *NAR5*^HapII^-GFP at 4 weeks post-inoculation with HH103; scale bar = 1 cm. **(G)** Immunoblotting analysis of GFP, *NAR5*^HapI^-GFP, and *NAR5*^HapII^-GFP protein levels in hairy roots, with actin as a loading control. Quantification of western blot bands grayscale values with actin calibration was performed via the native software package of the E-BLOT Touch Imager. **(H)** Relative expression level of GFP in **(F)**. Data are means ± SD (n = 3 biological replicates) and were compared using one-way ANOVAs with Tukey’s multiple-comparison test (*P* < 0.05), and different lowercase letters indicate significant differences among groups. **(I)** Nitrogenase activity levels in the nodules from **(F)**. Data are means ± SD (n = 10 biological replicates) and were compared using one-way ANOVAs with Tukey’s multiple-comparison test (*P* < 0.05), and different lowercase letters indicate significant differences among groups.


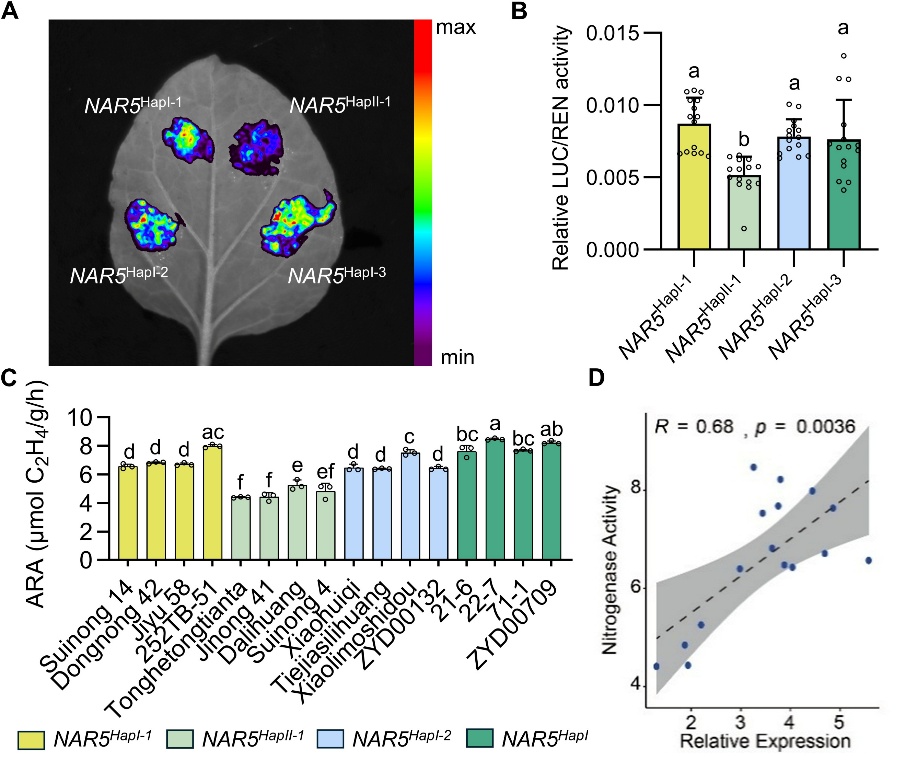


**Supplemental Figure 8. Natural *NAR5* promoter variation underlies differences in expression and impacts nitrogenase activity.**

**(A)** *NAR5*^HapI-1^, *NAR5*^HapII-1^, *NAR5*^HapI-2^, and *NAR5*^HapI-3^ haplotype promoter activity (3-kb upstream sequence) as analyzed via a dual-luciferase (LUC/REN) approach in tobacco leaves. **(B)** Quantification of the LUC/REN ratio from **(A)**. Data are means ± SD (n = 15 biological replicates). Different letters indicate statistically significant differences (*P* < 0.05, one-way ANOVAs with Tukey’s multiple-comparison test, and different lowercase letters indicate significant differences among groups). **(C)** Nitrogenase activity in root nodules from soybean accessions naturally harboring different NAR5 haplotypes. Data are means ± SD (n = 3 biological replicates), and were compared using one-way ANOVAs with Tukey’s multiple-comparison test (*P* < 0.05), different lowercase letters indicate significant differences among groups. **(D)** Correlations between *NAR5* expression levels and nitrogenase activity in *NAR5*^HapI-1^, *NAR5*^HapII-1^, *NAR5*^HapI-2^ and *NAR5*^HapI-3^ accessions.

**
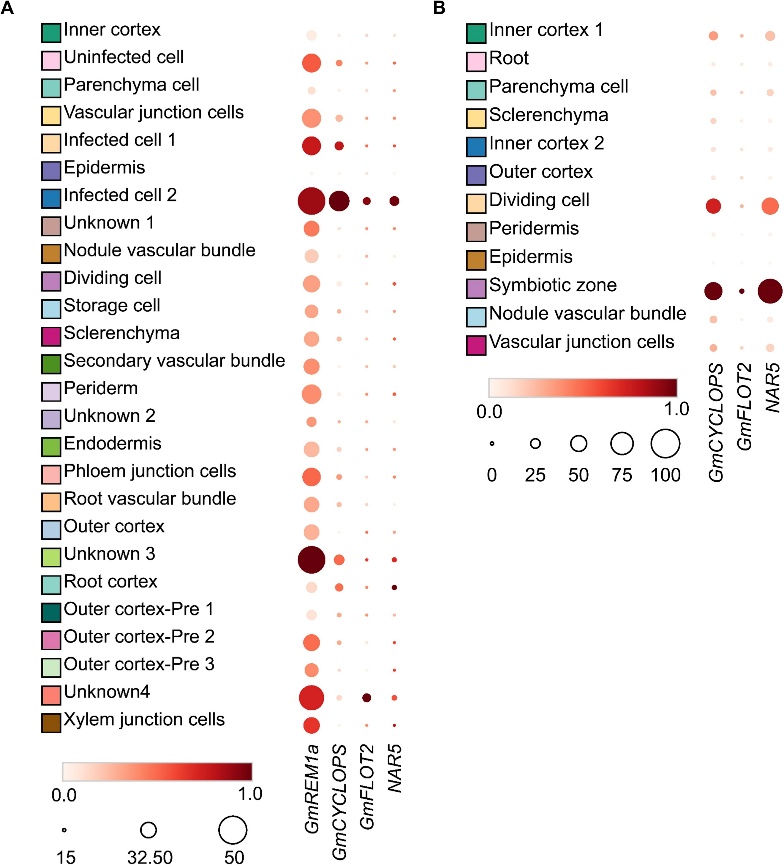
**

**Supplemental Figure 9. Cell-type-specific *NAR5* expression in infected cells from soybean nodules.**

**(A, B)** Single-cell RNA sequencing **(A)** and spatial transcriptomic analyses **(B)** revealing that *NAR5* is predominantly expressed in infected cells. *GmREM1*, *GmCYCLOPS* and *GmFLOT2* were used as infected cell-specific marker genes. Data were obtained from the SoyOmics database (https://ngdc.cncb.ac.cn/soyomics/index).


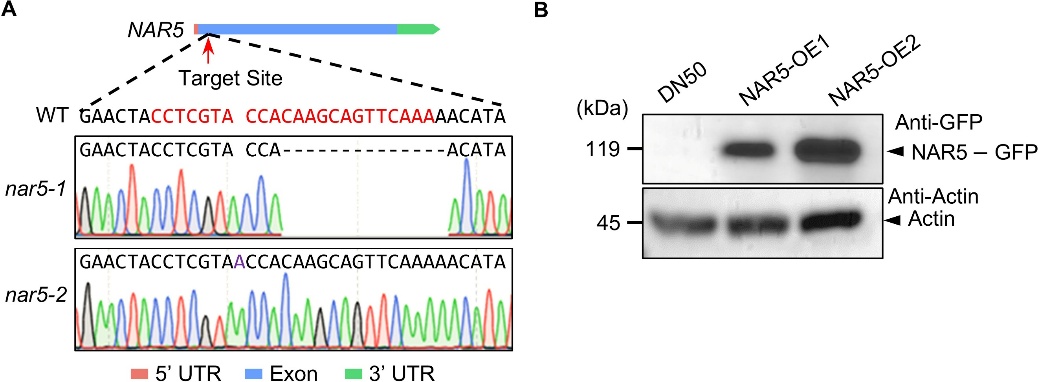


**Supplemental Figure 10. Molecular characterization of *nar5* mutants and transgenic *NAR5*-overexpressing lines.**

**(A)** *NAR5* gene editing using a CRISPR–Cas9 approach. Top: *NAR5* genomic structure, highlighting the sgRNA target site in an exon. Exons are represented by blue boxes, while UTRs are shown in red and green. Bottom: Sanger sequencing-based genotyping results for *nar5-1 and nar5-2* mutant alleles obtained in the DN50 background. **(B)** Immunoblotting analysis of *NAR5*-GFP protein levels in *NAR5*-overexpressing lines (*NAR5-OE1* and *NAR5-OE2*), with actin as a loading control.


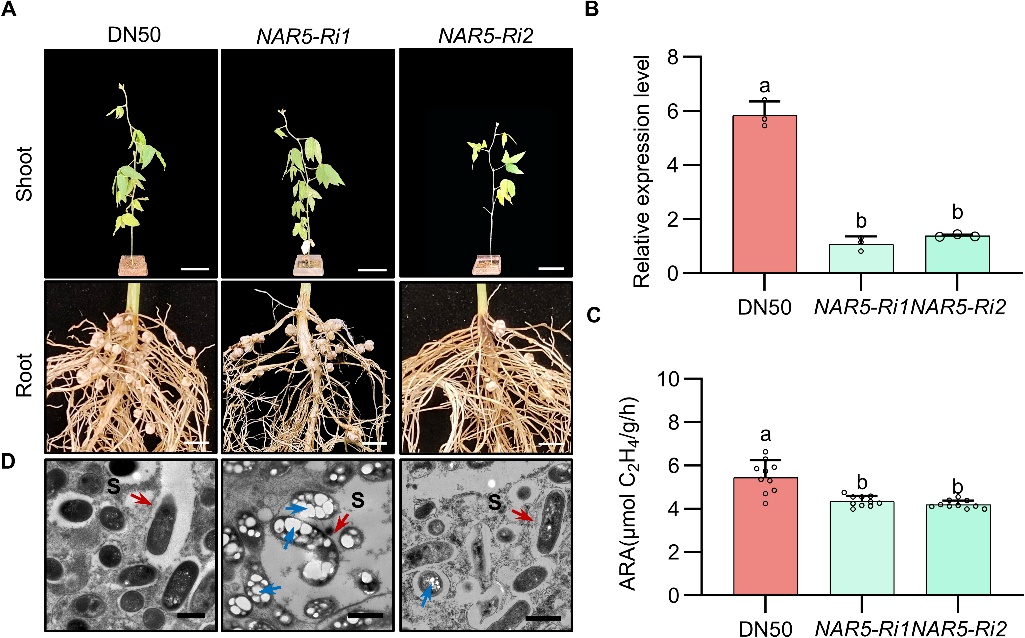


**Supplemental Figure 11. *NAR5* silencing results in reduced root nodule nitrogenase activity.**

**(A)** Root and shoot phenotypes from wild-type (DN50) and *NAR5*-silenced plants (*NAR5-Ri1* and *NAR5-Ri2*) at 4 weeks post-inoculation (wpi) with *S. fredii* HH103. Shoots: Scale bars = 7.5 cm; Roots: Scale bars = 5 mm. **(B)** *NAR5* expression in nodules from wild-type DN50 and the *NAR5-Ri1* and *NAR5-Ri2* silencing lines at 4 wpi with HH103. *UKN1* was used to normalize gene expression with the 2^-∆^*^C^*^T^ method. Data are means ± SD (n = 3 biological replicates), and were compared with one-way ANOVAs, different lowercase letters indicate significant differences among groups. **(C)** Analysis of the nitrogenase activity in **(A)**. Data are means ± SD (n = 10 biological replicates), and were compared using one-way ANOVAs with Tukey’s multiple-comparison test (*P* < 0.05), different lowercase letters indicate significant differences among groups. **(D)** Transmission electron micrographs of nodules from DN50 and *NAR5-Ri* plants at 4 wpi. S: Symbiosome and marked with red arrows. Poly-β-hydroxybutyrate (PHB) is indicated using blue. Scale bar = 1 μm.

**
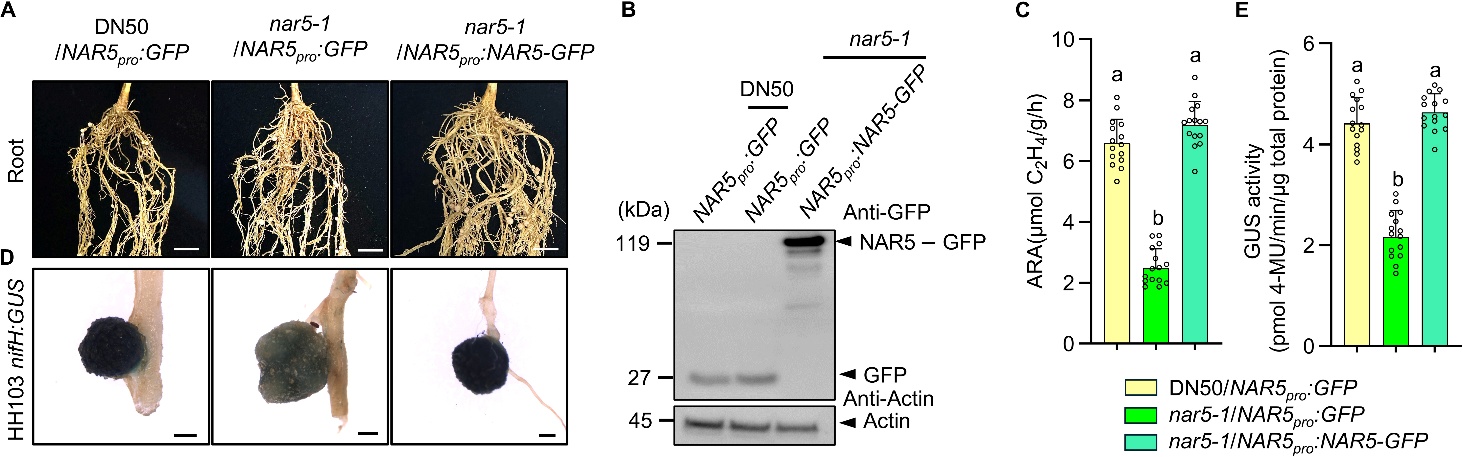
**

**Supplemental Figure 12. Impact of *NAR5-GFP* overexpression on nitrogenase activity in a *nar5-1* mutant.**

**(A)** Roots phenotypes in *nar5-1* hairy roots expressing GFP or *NAR5-GFP* under the control of the *NAR5* promoter from DN50 in the *nar5-1* or DN50, background at 4 weeks post-inoculation (wpi) with HH103. Scale bars = 1 cm. **(B)** Immunoblot of NAR5-GFP or GFP abundance in hairy roots. Actin was used as a loading control. **(C)** Quantification of the nitrogenase activity in **(A)**. Data are means ± SD (n = 10 biological repeats) and were compared using one-way ANOVAs with Tukey’s multiple-comparison test (*P* < 0.05), different lowercase letters indicate significant differences among groups. **(D)** Images of mature nodules from hairy roots expressing GFP or *NAR5-GFP* in the *nar5-1* or DN50 background at 4 wpi with HH103 *nifH:GUS*. Scale bars = 1 mm. **(E)** Quantification of GUS enzyme activity in **(D)**. Data are means ± SD (n = 10 biological replicates) and were compared using one-way ANOVAs with Tukey’s multiple-comparison test (*P* < 0.05), different lowercase letters indicate significant differences among groups.


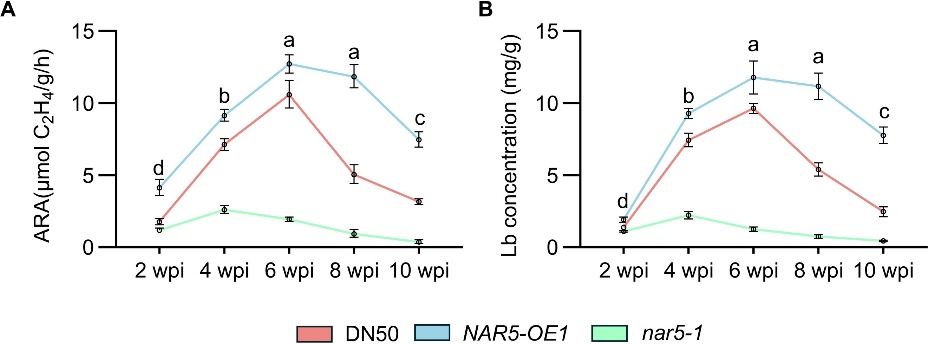


**Supplemental Figure 13.** **Nitrogenase activity and leghemoglobin content in DN50, *NAR5-OE1*, and *nar5-1* root nodules over time.**

**(A, B)** Nitrogenase activity **(A)** and Leghemoglobin (Lb) content **(B)** in DN50, *NAR5-OE1,* and *nar5-1* root nodules at 2, 4, 6, 8, and 10 wpi. Red, light blue, and green lines respectively correspond to DN50, *NAR5-OE1*, and *nar5-1*. Data are means ± SD (n = 3 biological replicates), and were compared using one-way ANOVAs with Tukey’s multiple-comparison test (*P* < 0.05), different lowercase letters indicate significant differences among groups.

**
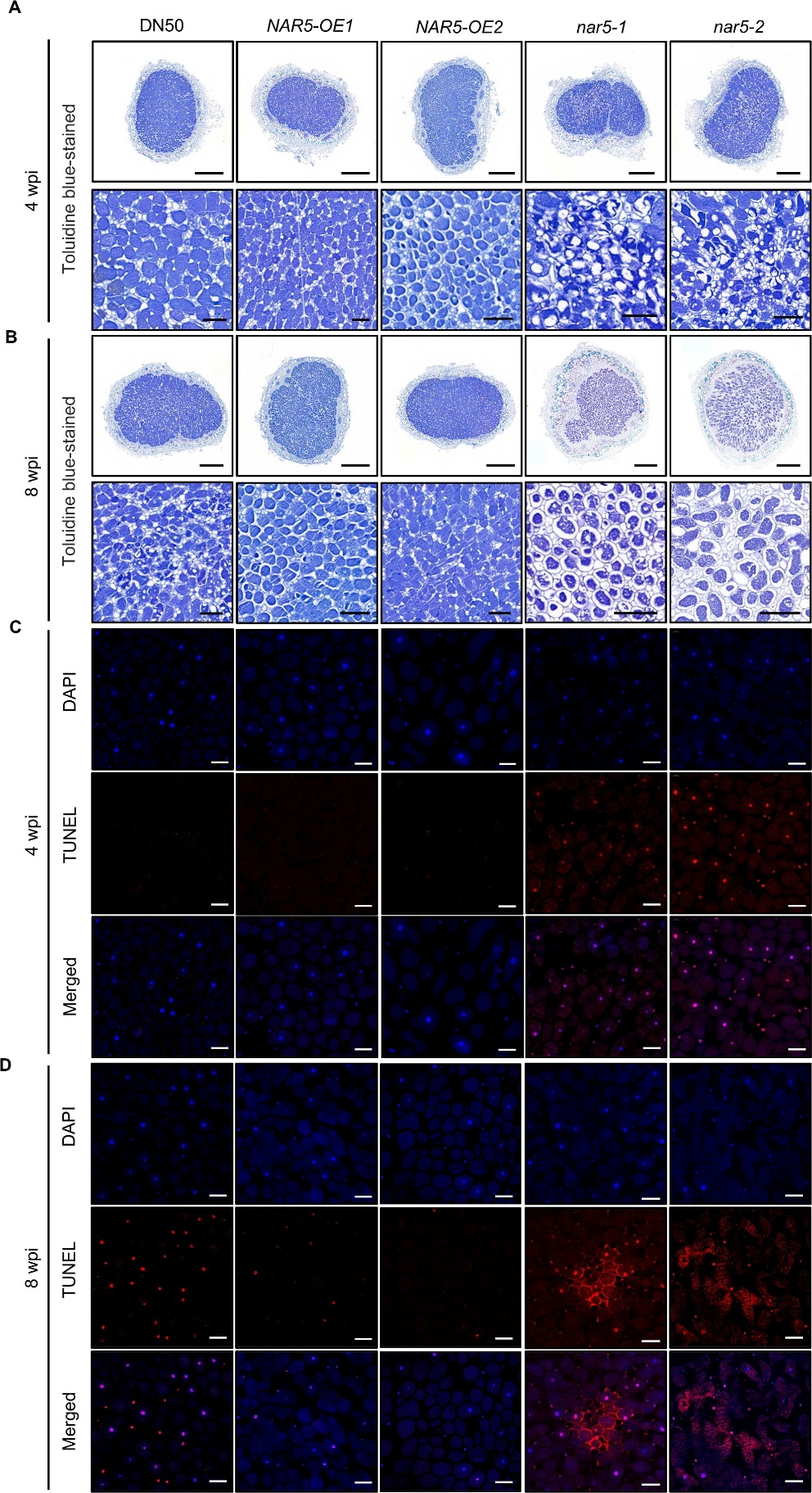
**

**Supplemental Figure 14. NAR5 inhibits nodule senescence.**

**(A, B)** Longitudinal root nodule sections from plants of the indicated genotypes following toluidine blue staining at 4 wpi (A) and 8 wpi **(B)**. Upper panels: scale bars = 1 mm; Lower panels: scale bars = 100 µm. **(C, D)** TUNEL staining for apoptotic activity in nodules at 4 wpi **(C)** and 8 wpi **(D)**. Images show nuclei and rhizobia (DAPI, upper), DNA fragmentation (TUNEL, middle), and merged overlay images (lower). Scale bars = 50 µm. Representative images are shown from at least 5 independent embedded samples.


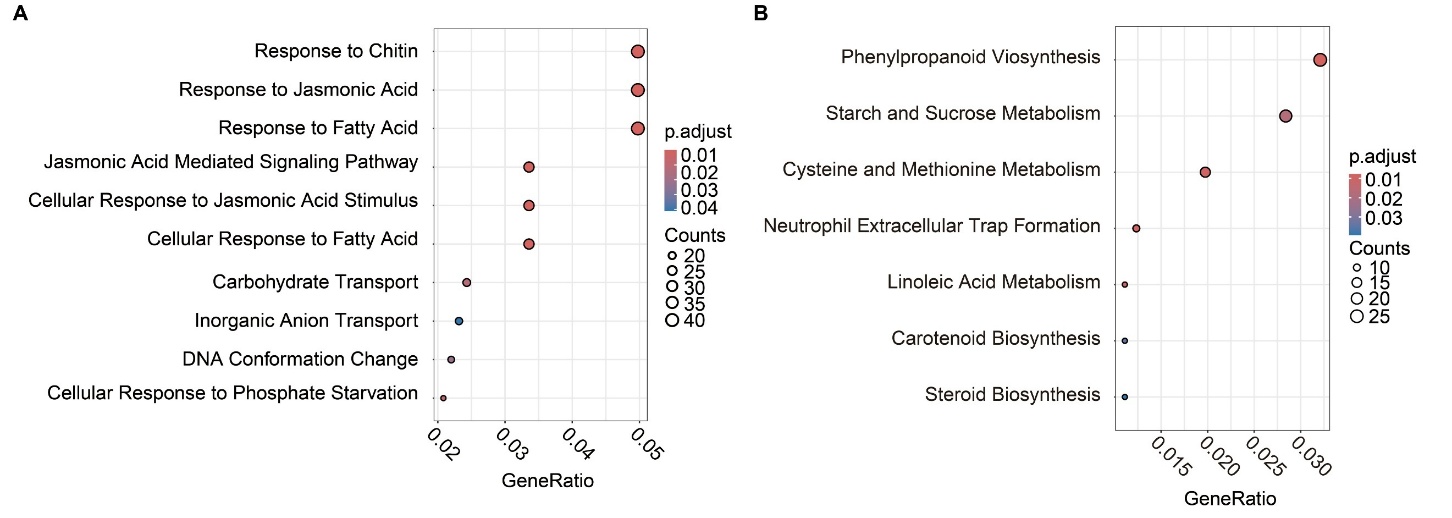


**Supplemental Figure 15. Gene Ontology and KEGG enrichment analyses of differentially expressed genes.**

**(A, B)** Gene Ontology **(A)** and KEGG enrichment **(B)** analysis of common differentially expressed genes (DEGs) identified when comparing *NAR5-OE1* with DN50 at 4 wpi and 8 wpi.


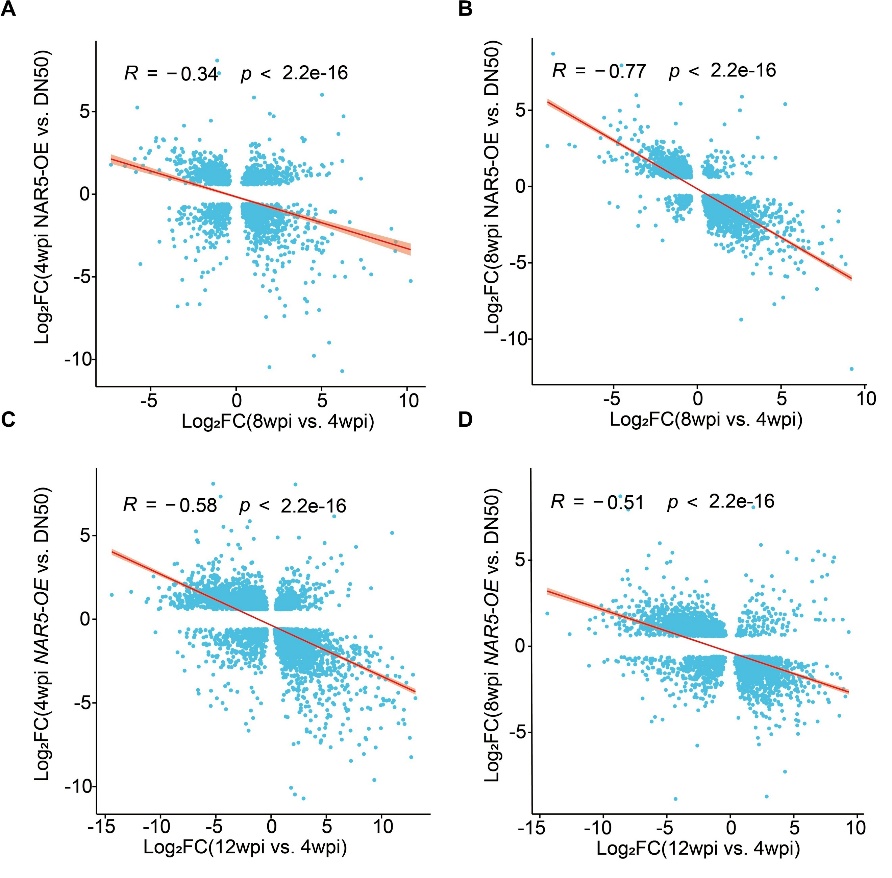


**Supplemental Figure 16. Transcriptional relationship between *NAR5* overexpression and nodule senescence.**

**(A, B)** Correlation scatterplots depicting the association between DEGs from *NAR5-OE1* and DEGs from the comparison of nodules at 8 wpi vs. 4 wpi (representing early aging) at 4 wpi **(A)** and 8 wpi **(B)**. **(C, D)** Correlation scatterplots depicting the association between of DEGs from *NAR5-OE1* and DEGs from the comparison of 12 wpi vs. 4 wpi nodules (representing advanced aging) at 4 wpi **(C)** and 8 wpi **(D)**. The linear regression results are shown with a red line. *R*, Pearson correlation coefficient. FC, Fold change. RNA-seq data from healthy nodules (at 4 wpi) and senescent nodules (at 8 wpi and 12 wpi) were downloaded from a prior publication (Yu et al., *The Plant Cell*. Data. 2023).

**
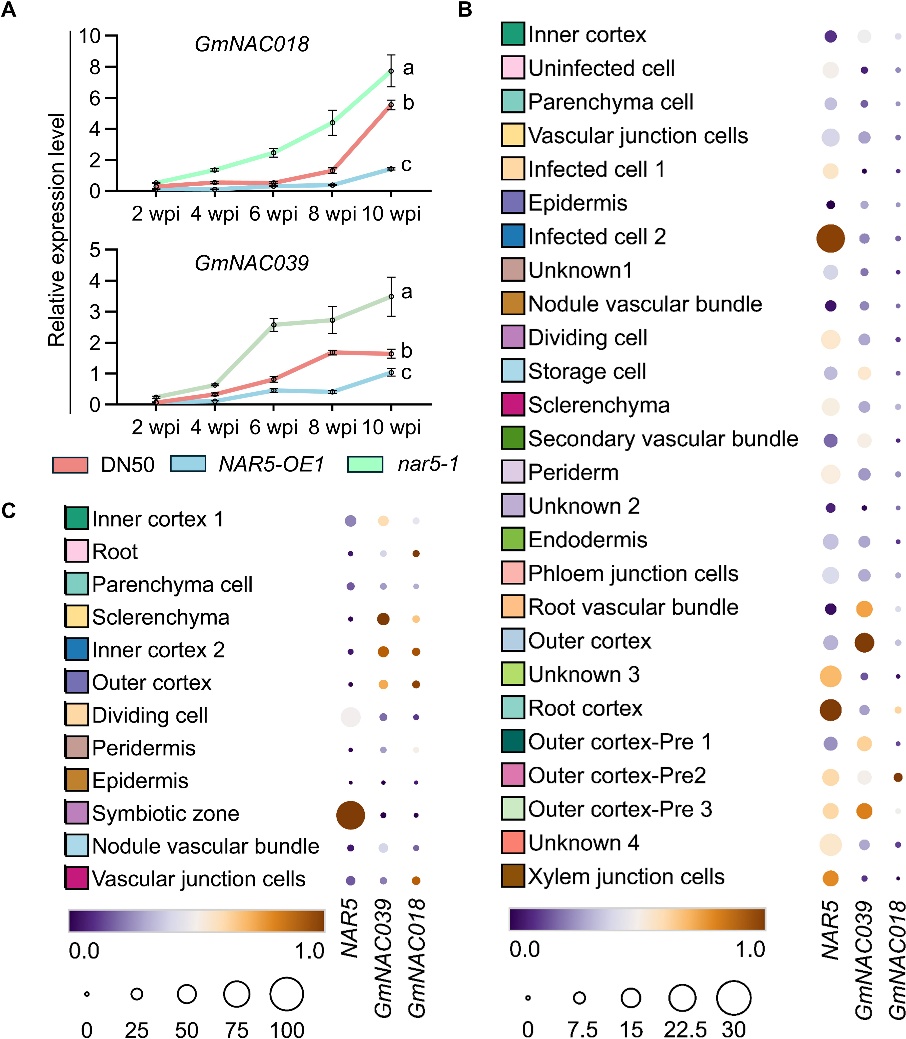
**

**Supplemental Figure 17. Spatiotemporal expression profiling of *GmNAC039* and *GmNAC018*.**

**(A)** Relative expression of *GmNAC018* and *GmNAC039* in DN50, *NAR5-OE1* or *nar5-1* mutant nodules at 2, 4, 6, 8, and 10 wpi. Red, light blue, and green lines respectively correspond to DN50, *NAR5-OE1* and *nar5-1* mutant. Data are means ± SD (n = 3 biological replicates), and were compared using two-way ANOVAs with Tukey’ s multiple-comparison test (*P* < 0.05), different lowercase letters indicate significant differences among groups. **(B and C)** Single-cell RNA sequencing **(B)** and spatial transcriptomic analyses **(C)** revealing the cell-type specificity and spatial patterning of *GmNAC039* and *GmNAC018* expression.  Data were obtained from the SoyOmics database (https://ngdc.cncb.ac.cn/soyomics/index).

**
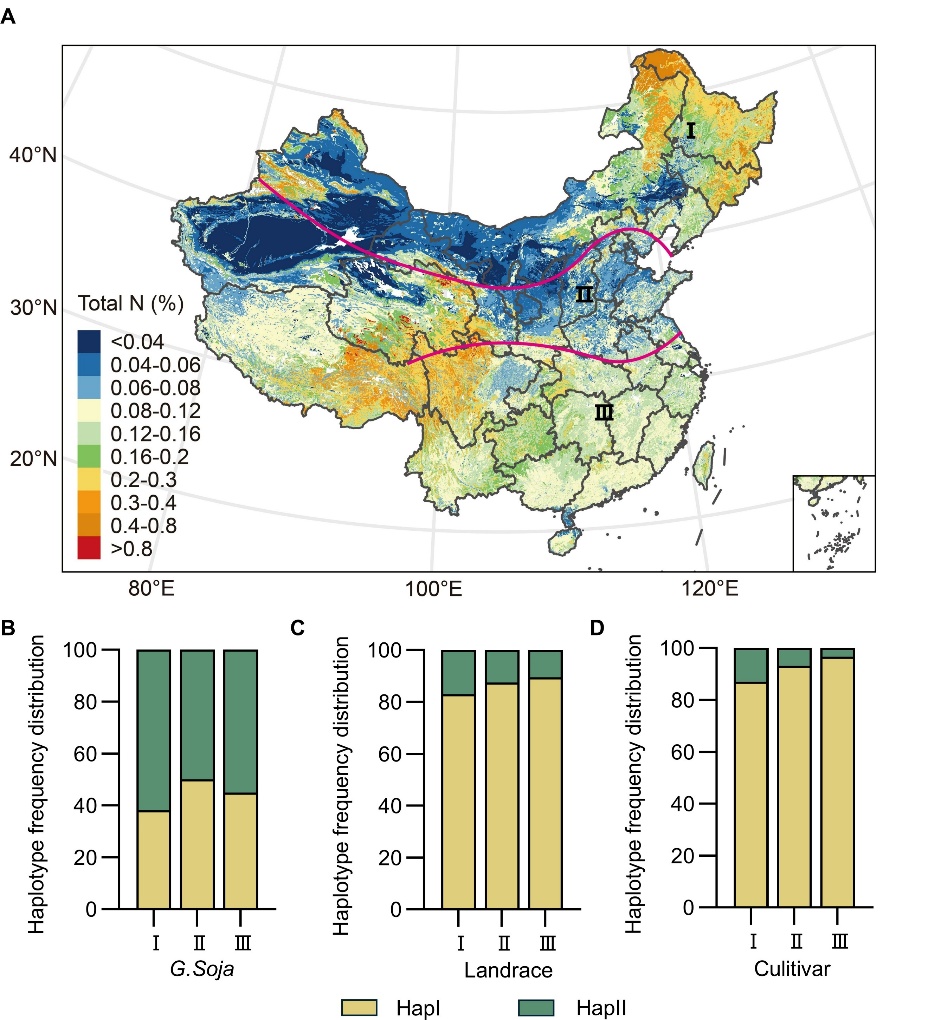
**

**Supplemental Figure 18. Geographic distribution of the *NAR5*^HapI^ and *NAR5*^HapII^.**

**(A)** Total plow layer (0 – 30 cm) nitrogen content across China. Data were sourced from a prior publication (Shangguan et al., *Journal of Advances in Modeling Earth Systems*. 2014). **(B–D)** *NAR5* haplotype distribution across China, including Ecoregion I (n = 1,003 accessions), Ecoregion II (n = 768 accessions), and Ecoregion III (n = 620 accessions).


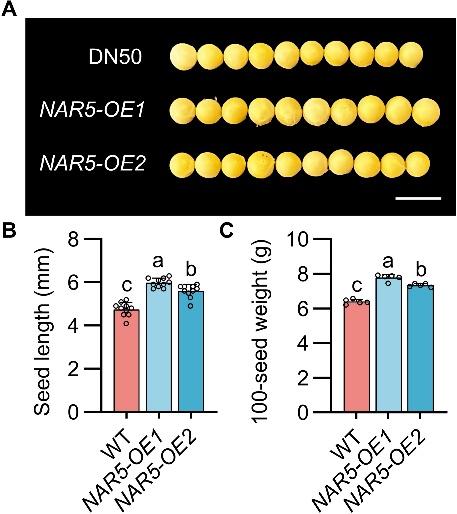


**Supplemental Figure 19. *NAR5* overexpression enhances soybean yield-related traits.**

**(A)** Seed width images for DN50 and *NAR5-OE* lines. Scale bars = 1 cm. **(B)** Quantification of the seed widths in **(A)**. Data are means ± SD (n = 10 biological replicates) and were compared using one-way ANOVAs with Tukey’s multiple-comparison test (*P* < 0.05), different lowercase letters indicate significant differences among groups. **(C)** 100-seed weight values for DN50 and *NAR5-OEs*. Data are means ± SD (n = 10 biological replicates) and were compared using one-way ANOVAs with Tukey’s multiple-comparison test (*P* < 0.05), different lowercase letters indicate significant differences among groups.


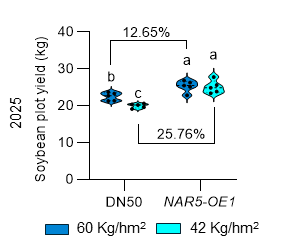


**Supplemental Figure 20. *NAR5* overexpression enhances plot yield and adaptability to low-nitrogen conditions in 2025**

Plot yield of *NAR5-OE1* and *DN50* plant per plot (~30 m^2^) in 2025 field season, under standard (60 kg N ha⁻¹) or low-nitrogen (30% less, 42 kg N ha⁻¹) treatments (n = 5 plots). Data were compared using two-way ANOVAs with Tukey’s multiple-comparison test (*P* < 0.05), and different lowercase letters indicate significant differences among groups.

**Supplementary Tables**

**Supplemental Table 1** Phenotypic statistics of nitrogen fixation activity in 309 soybean accessions and RIL population

**Supplemental Table 2** Significant SNP on Chromosome 5 using MLM model

**Supplemental Table 3** Marker information within candidate QTL regions

**Supplemental Table 4** Differentially expressed genes identified using RNA-seq of *NAR5-OE1* vs. DN50 at 4 wpi

**Supplemental Table 5** Differentially expressed genes identified using RNA-seq of *NAR5-OE1* vs. DN50 at 8 wpi

**Supplemental Table 6** Sequence information of *NAR5* haplotypes.

**Supplemental Table 7** Chemical properties of the top 25-cm soils from the experimental area (n = 5). Mean ± SD
